# Supplementary material for: SOTIP is a versatile method for microenvironment modeling with spatial omics data
Source: Nat Commun. 2022 Nov 28;13:7330. doi: 10.1038/s41467-022-34867-5 (PMC9705407; doi:10.1038/s41467-022-34867-5)
Supplement: Supplementary file 1 — Supplementary Figures Tables and Notes [file 41467_2022_34867_MOESM1_ESM.pdf]

Supplementary Figure 1

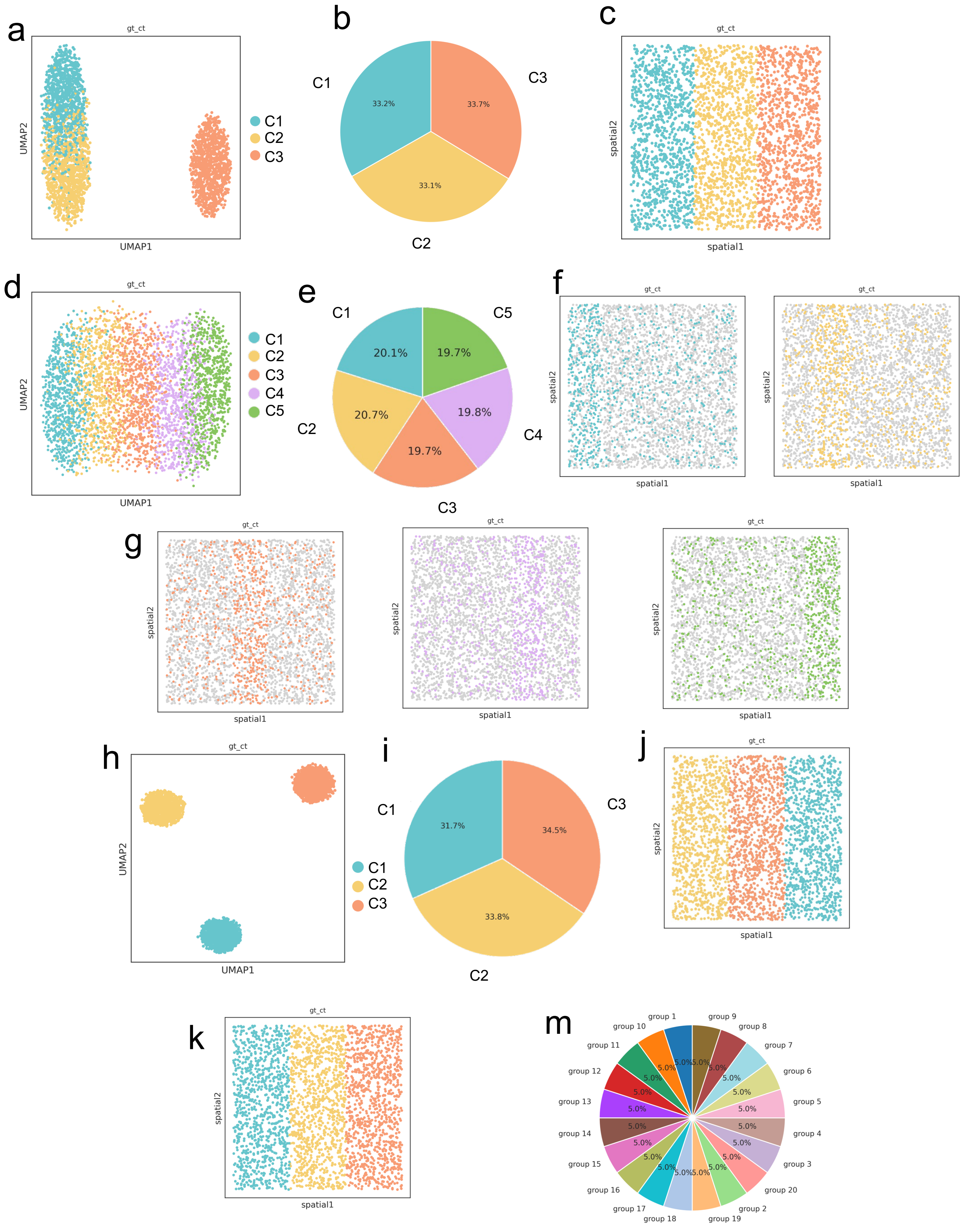

Supplementary Figure 1. Information of simulation data.

Legend in next page…

Supplementary Figure 1. Information of simulation data.
a-c, UMAP plot (a), cell composition (b), and spatial distribution (c) of simulation 1 with the same color scheme.
d-g, UMAP plot (d), cell composition (e), and spatial distribution (f-g) of simulation 2 with the same color scheme.
h-k, UMAP plot (h), cell composition (i), and spatial distribution (j-k) of simulation 3 with the same color scheme.
m, Cell composition of Simulation 4.

Supplementary Figure 2

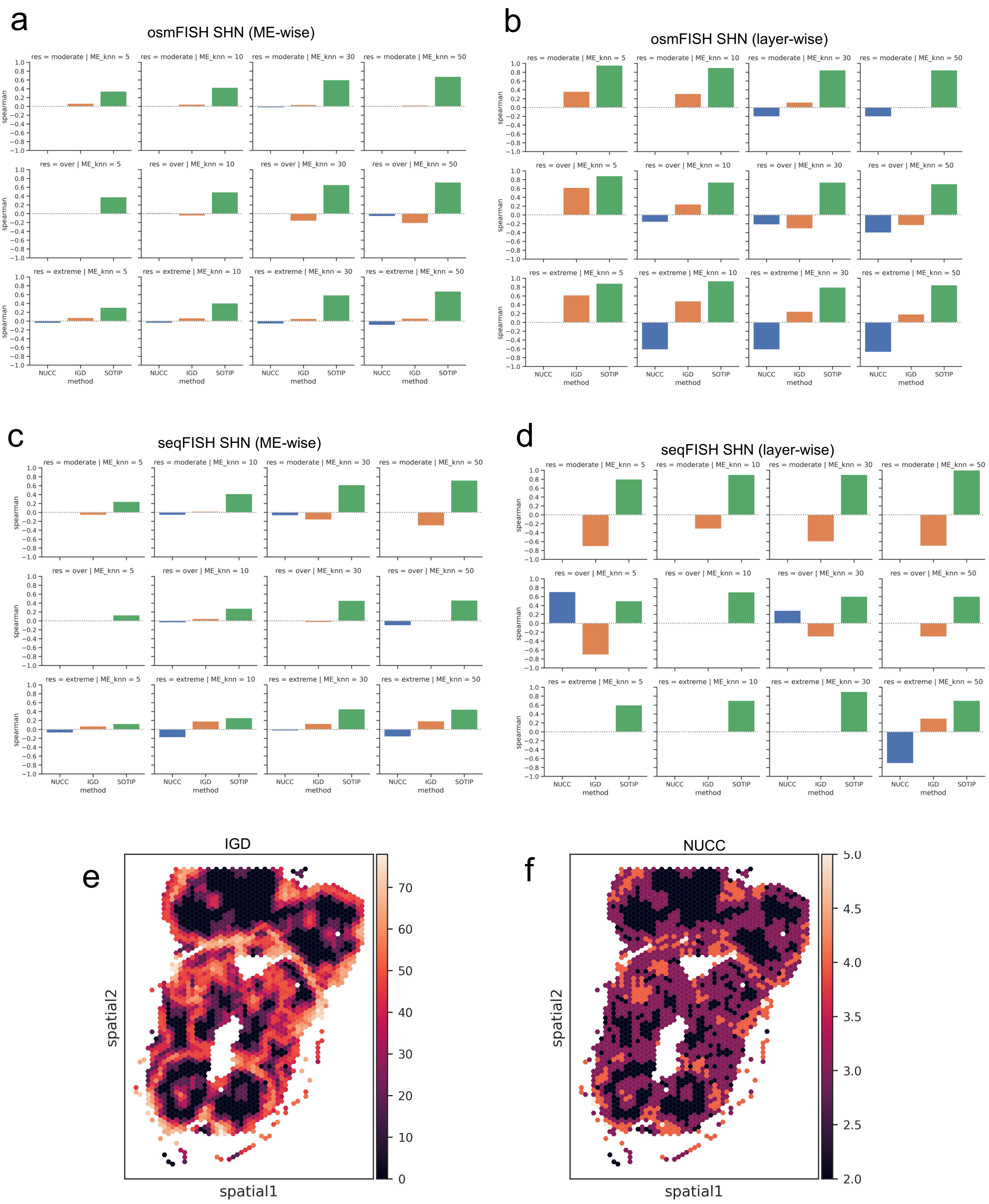

Supplementary Figure 2. Associated with Figure 3.

Legend in next page…

Supplementary Figure 2. Associated with Figure 3.

a, ME-wise correlation between SHNs and cortical depth with different combinations of parameters in SHN quantification. There are 3 rows and 4 columns. Each of the three row corresponds to a clustering resolution, and each column uses a ME_knn parameter (kNN of the ME size). The colors of bar plots represent different compared methods.
b, Same with a, but using layer-wise correlation.
c-d, similar with a-b, but on seqFISH cortex data.
e, 10x Visium spots paired with Fig. 3g, colored by IGD computed SHN.
f, 10x Visium spots paired with Fig. 3g, colored by NUCC computed SHN.

Supplementary Figure 3

a

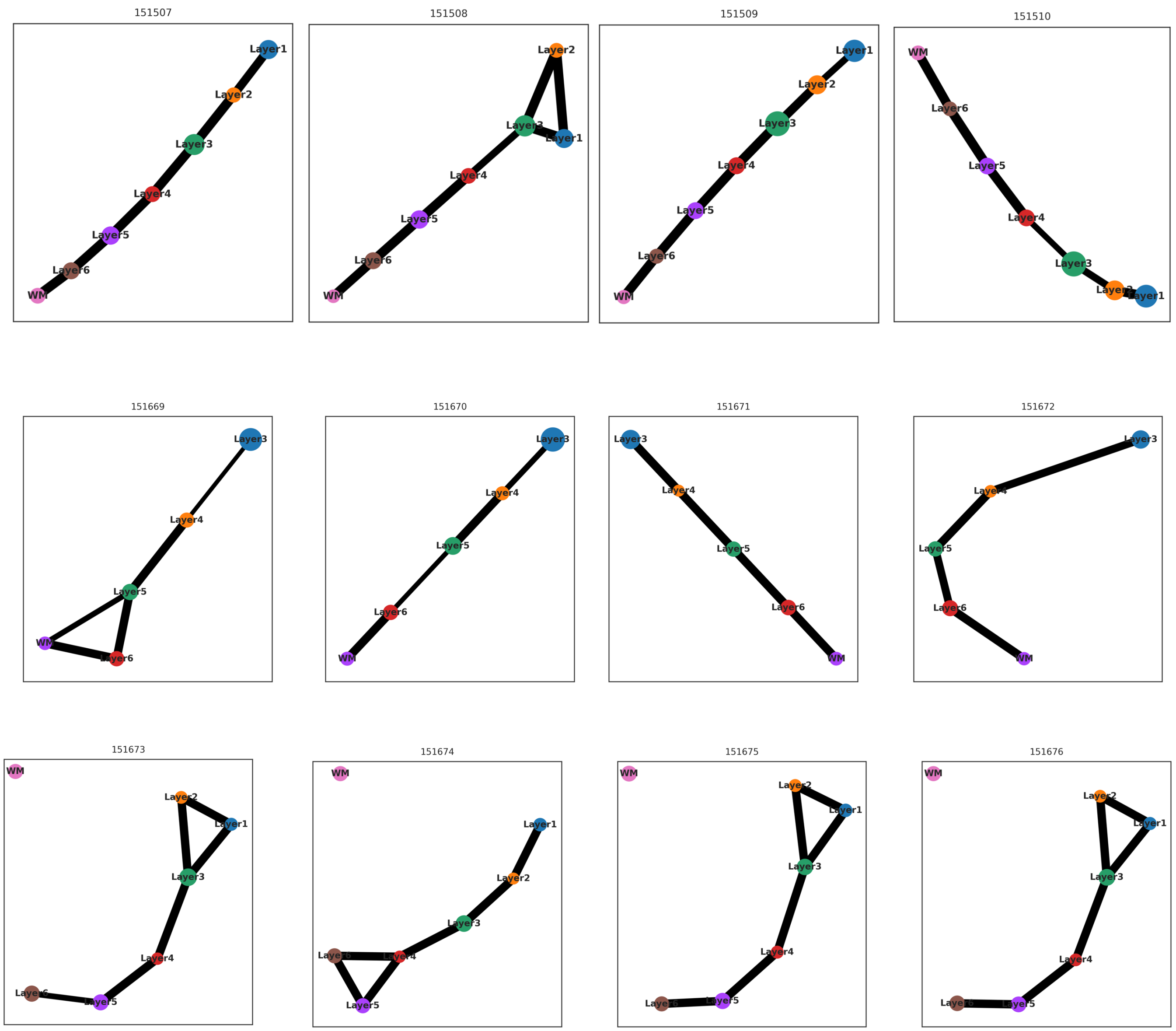

Supplementary Figure 3. Spatial trajectory inference on SpatialLIBD.
a, Spatial trajectory inference 12 samples of SpatialLIBD dataset. In each plot, each point is a tissue region (i.e., cortex layer), the edge width between nodes is proportional to the connectivity, edges associated with low connectivity is omitted for visualization.

Supplementary Figure 4

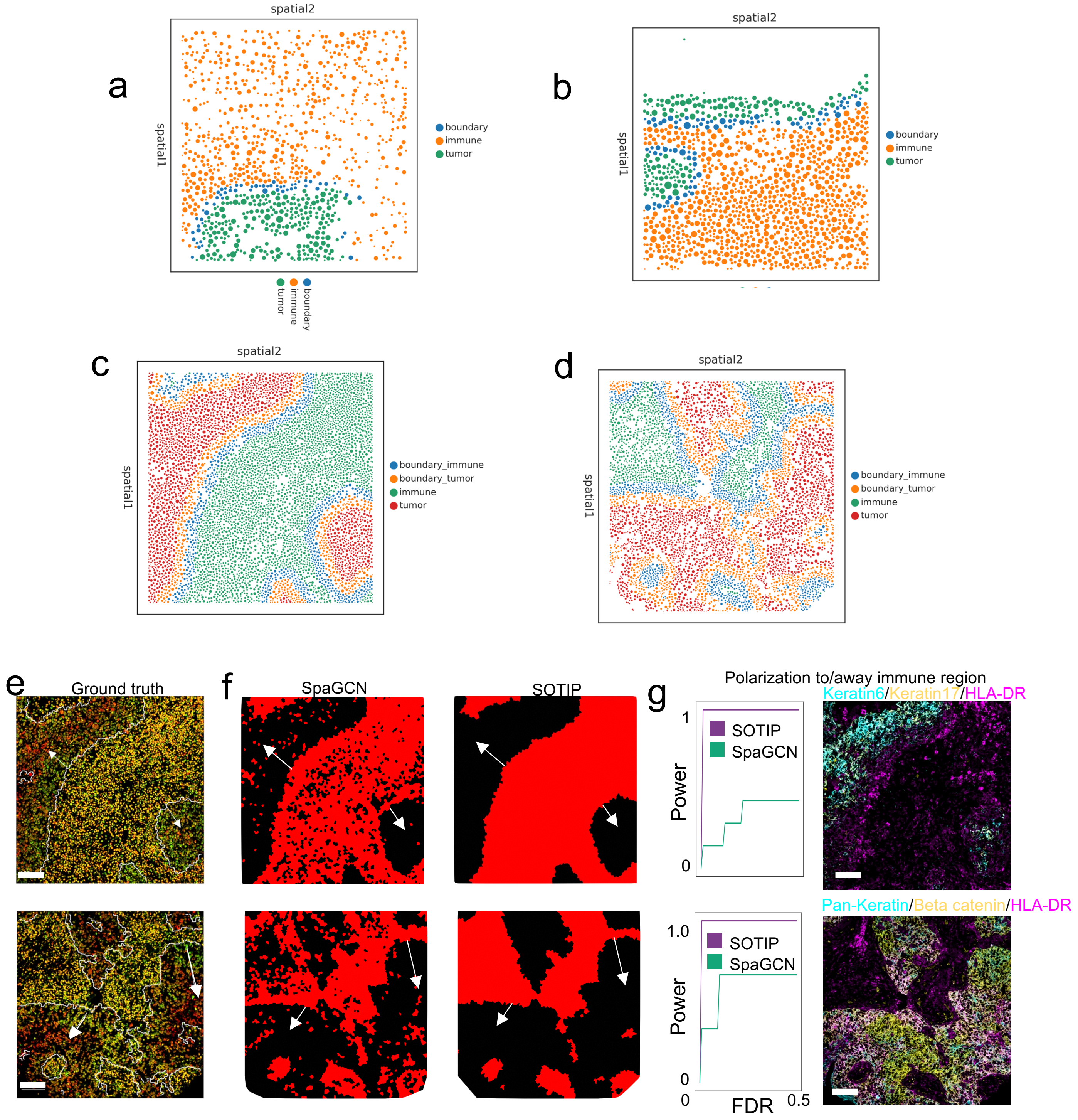

Supplementary Figure 4. Associated with Figure 5.

Legend in next page…

Supplementary Figure 4. Associated with Figure 5.

For brevity, the brackets represent the labels of the legend in figure.
a-b, Classification of cells into 3 categories: tumor region (labeled as tumor), immune distal to tumor region (labeled as boundary), and immune proximal to tumor region (labeled as immune), for point 16 (a) and point 23 (b). Dot size is proportional to cell size.
c-d, Classification of cells into 4 categories: tumor proximal to immune region (labeled as boundary_tumor), tumor distal to immune region (labeled as tumor), immune proximal to tumor region (labeled as boundary_immune), and immune distal to tumor region (labeled as immune), for patient 4 (c) and patient 9 (d). Dot size is proportional to cell size.
e-f, The same as Fig. 5g-h. For the convenience of comparison with Supplementary Fig. 4g. Scale bar: 100um.
g, Comparison of SpaGCN-detected and SOTIP-detected spatial domains to find polarized proteins to/away immune region, in TNBC patient 4 (top row) and TNBC patient 9 (bottom row). The left column shows that the power plots show the proportion of true positives (y axis) detected by different methods at a range of FDRs (x axis) for patient 4 (top row) and patient 9 (bottom row). The right column shows the overlay of 3 representative polarized proteins find by SOTIP-detected SDM in point 4 (top row) and point 9 (bottom row). Scale bar: 100um.
The experiment results in (e,g) were similar with 3 independent repeats.

Supplementary Figure 5

a

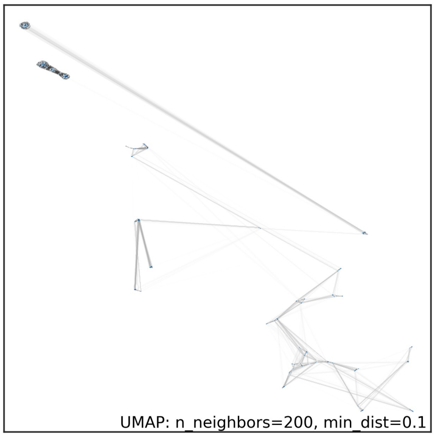

Supplementary Figure 5. Associated with Figure 6.
a, The original MECN graph of joint analysis of fibrotic and healthy liver samples. Each node is a MECN, and the edge between two nodes is the connectivity.

Supplementary Figure 6

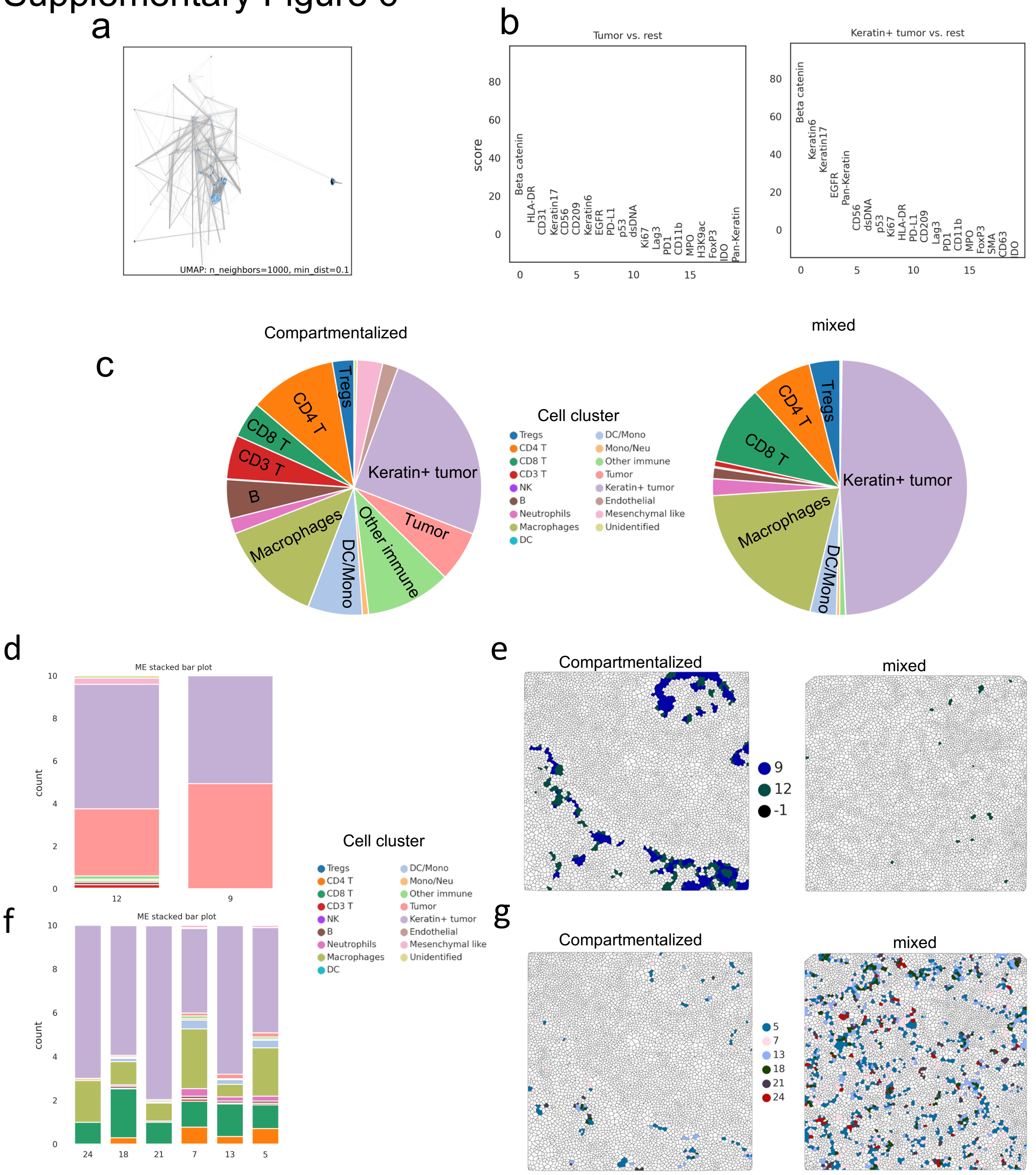

Supplementary Figure 6. Associated with Figure 7.

Legend in next page…

Supplementary Figure 6. Associated with Figure 7.

a, The original MECN graph of joint analysis of two TNBC samples. Each node is a MECN, and the edge between two nodes is the connectivity.
b, Top marker proteins of Tumor and Keratin+ tumor clusters. Score computed with SCANPY default parameter of function sc.tl.rank_gene_groups.
c, Cell composition of *compartmentalized* (left) and *mixed* (right) samples of TNBC, with the same color scheme as Fig. 7b.
d-g, Differential microenvironments of compartmentalized (d,e) and mixed (f,g) with 0.5 EMC cutoff.
d, Cell type composition of MECN cluster 12 and 9, which were more enriched in compartmentalized sample.
e, The spatial distribution of MECN cluster 12 and 9 on both samples.
f, Cell type composition of MECN cluster 24, 18, 21, 7, 13, 5 which were more enriched in mixed sample.
g, The spatial distribution of MECN cluster 24, 18, 21, 7, 13, 5 on both samples.

Supplementary Figure 7

a

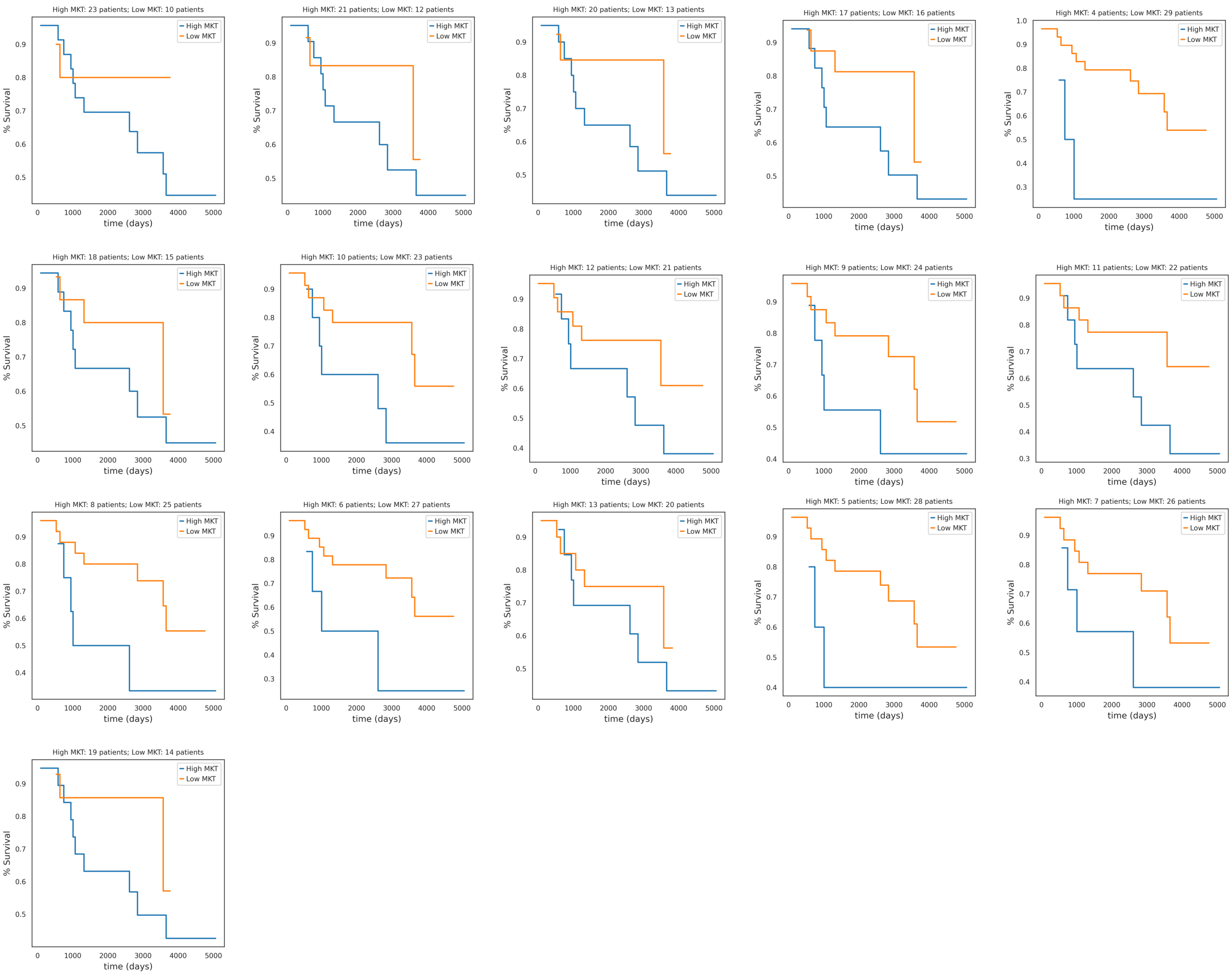

Supplementary Figure 7. Associated with Figure 7n.
a, Multiple choice of MKT occurrence score for patient grouping in survival analysis.

Supplementary Figure 8

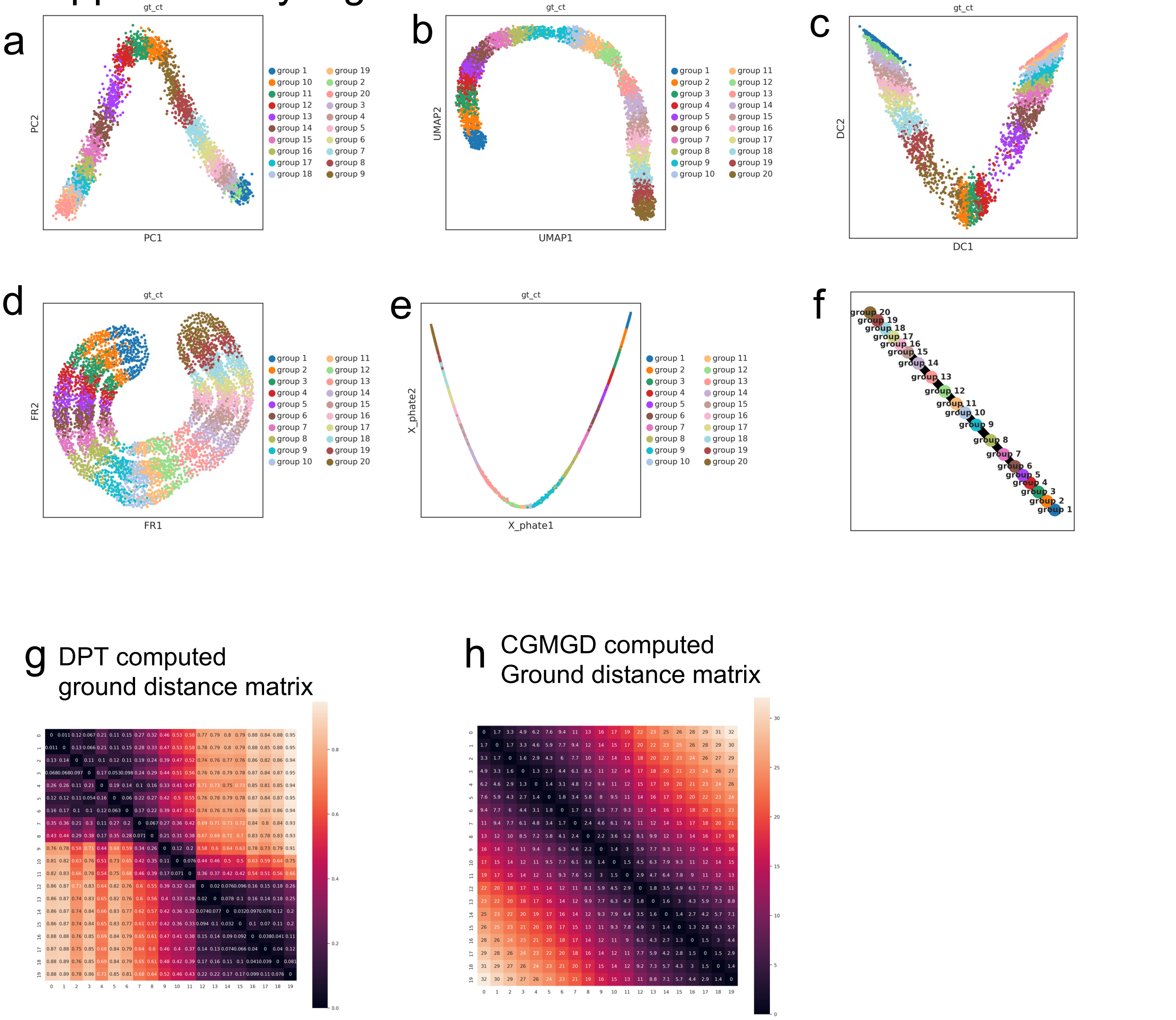

Supplementary Figure 8. Evaluation of ground distance.

With simulation 4 (see Methods), we attempted to use different embedding methods to compute the distance in the embedded space as the ground distance of earth mover's distance, which is further used to construct the MECN graph. These embedding methods include most popular ones, such as PCA (a), UMAP (b), diffusion map (c), ForceAtlas (d), and PHATE (e). All these embedding methods bended the simulated manifold of cells, thus leading to simply utilizing Euclidean distance problematic. The PAGA methods only capture the connectivity between closely related clusters, and simply treat far clusters disconnect (f). We also extended the diffusion pseudo-time (DPT) method by iterating DPT by assigning the root cell to each cluster center. This practice is time consuming, and fails to obtain comparable distance values obtained by each run of DPT (g). Our proposed CGMGD method best approximated the mutual manifold distance between cell clusters (h).

Supplementary Figure 9

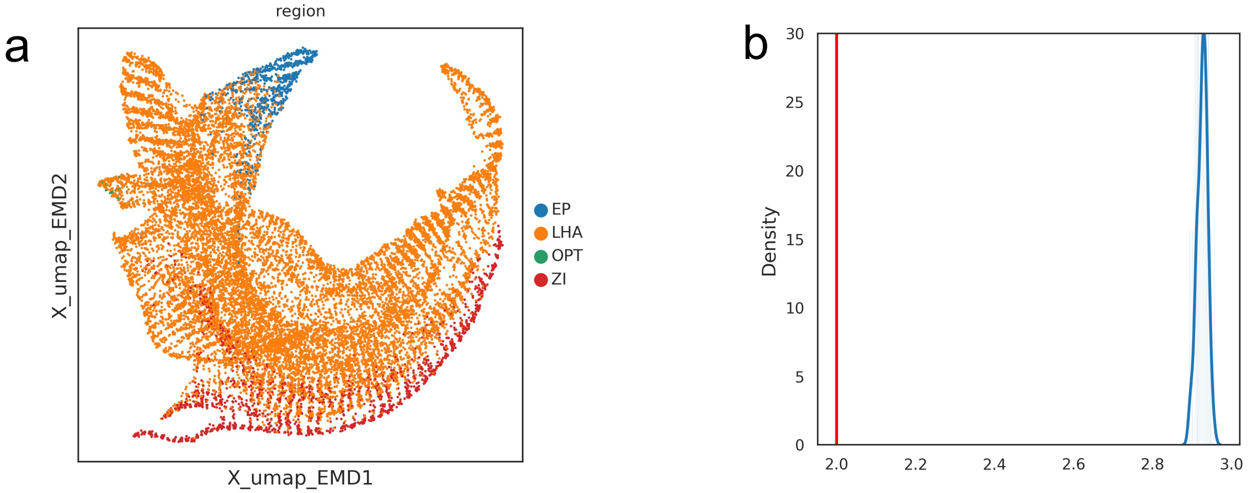

Supplementary Figure 9. SOTIP application on EASI-FISH dataset.
a, The MECN UMAP embedding computed by MEG. Each point is a MECN colored by anatomical annotations.
b, Blue curve: WB ratio (the Within-cluster-Between-cluster ratio) background distribution computed by random label permutation (n=100). Red line: observed WB ratio.

Supplementary Figure 10

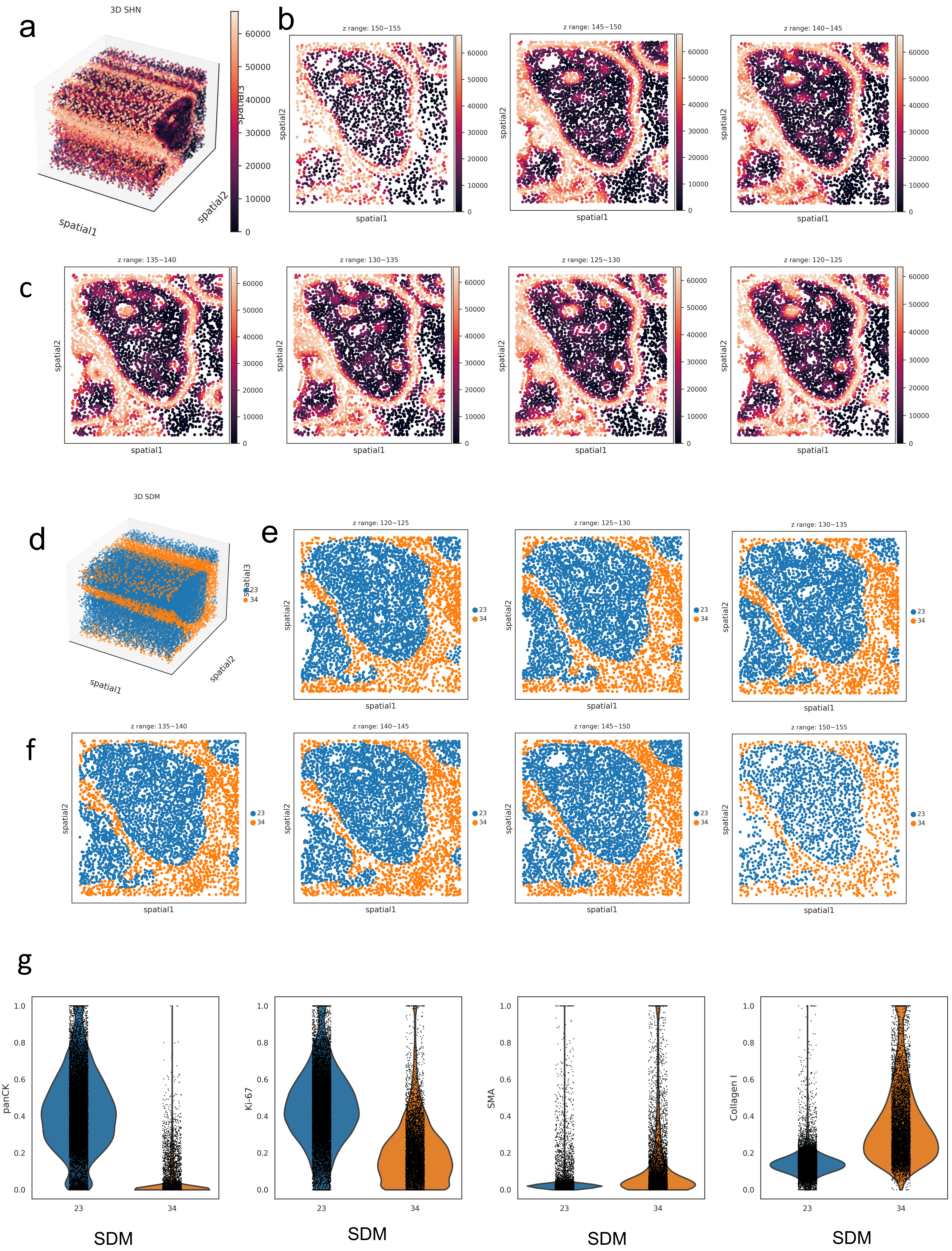

Supplementary Figure 10. SOTIP application on 3D IMC dataset.
Legend in next page…

Supplementary Figure 10. SOTIP application on 3D IMC dataset.
a-c, SHN performed by SOTIP shown in 3D (a) and in silico sliced sections along z-axis (b-c).
d-f, SDM performed by SOTIP shown in 3D (d) and in silico sliced sections along z-axis (e-f).
g, SOTIP successfully stratifies different tissue regions (i.e. tumor and stroma region), consistent with the marker enrichment.

Supplementary Figure 11

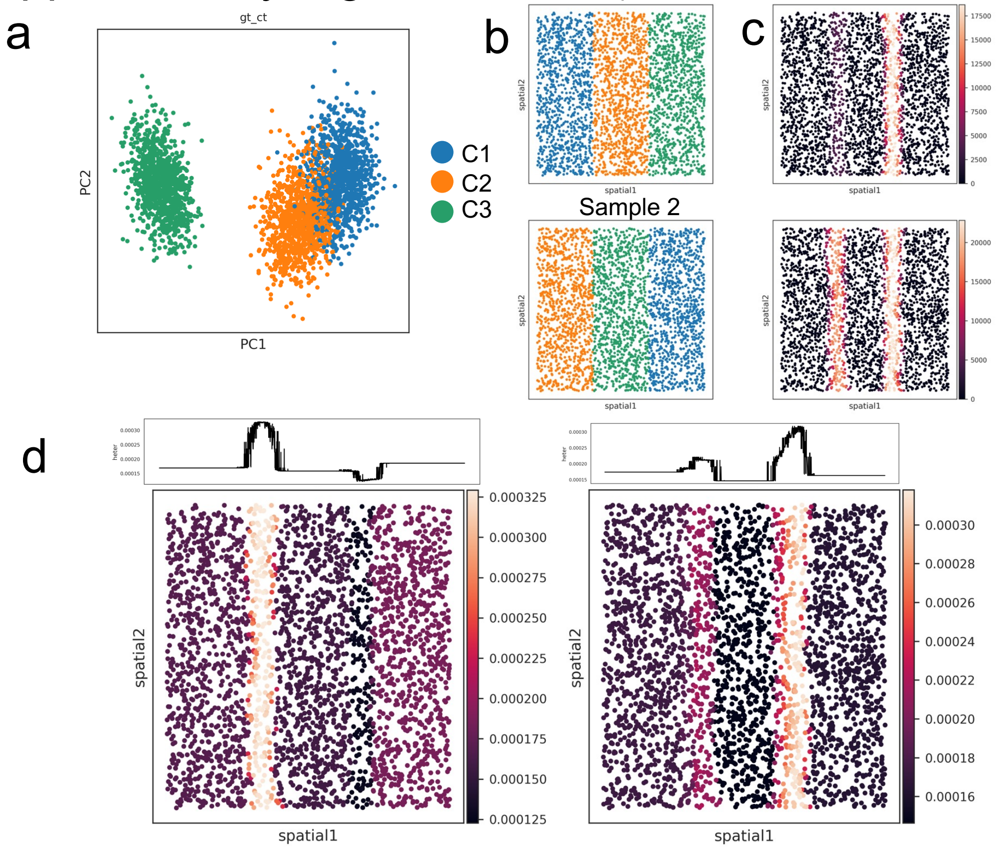

Supplementary Figure 11. Simulation 5.
This simulation is similar with Fig. 2e-f, in which the aim is to detect sample specific boundaries. The difference is that, in simulation 3 (Fig. 2e-f), the three cell clusters share the same distance in gene expression manifold, while in simulation 5 (this figure), the C1-C2 are closer, while C3 is further from C1 and C2 (a).
(b): Sample 1 (upper panel) and sample 2 (lower panel) are formed by two different spatial distribution of cells in (a).
(c): The SHN computed by SOTIP in sample 1(upper panel) and sample 2 (lower panel).
(d): Sample-specific microenvironments (left panel for sample 1, right panel for sample 2) are highlighted by MEG constructed with SOTIP. Each panel consists of two parts. Take the left panel as an example, the bottom part shows the same set of cells as in (b), colored according to the relative likelihood of observing each microenvironment in sample 1, and the top part shows the value of relative likelihood of each MECN as a function of horizontal coordinate.

Supplementary Figure 12

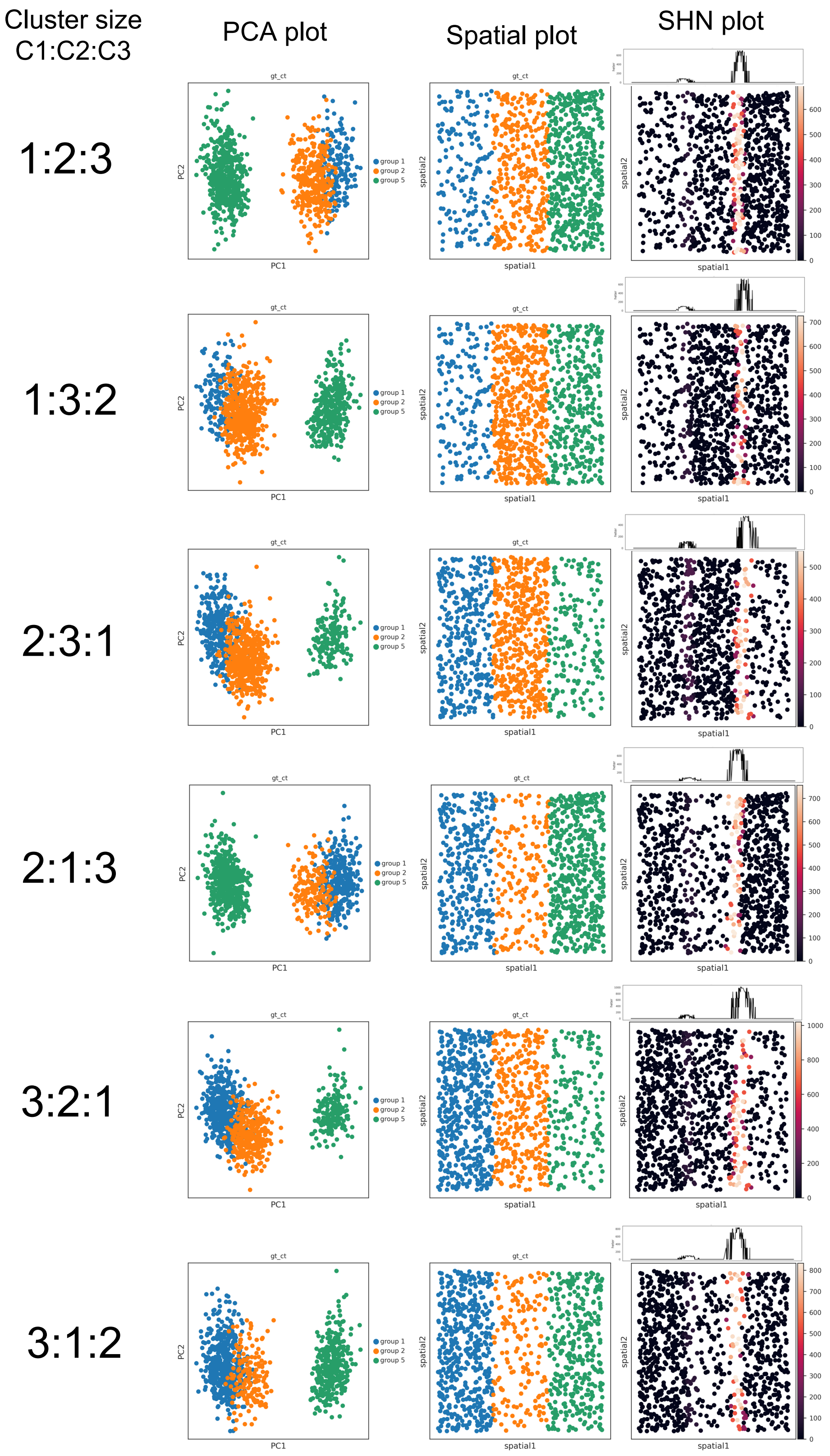

Supplementary Figure 12. Simulation 6.
This simulation shows the robustness of SOTIP-SHN module given different cluster size. There are 6 rows in this figure, and each row shows the cluster size ratio (column 1), PCA plot (column 2), spatial distribution of cells (column 3), and spatial plot colored by SHN values computed by SOTIP (column 4).

Supplementary Figure 13

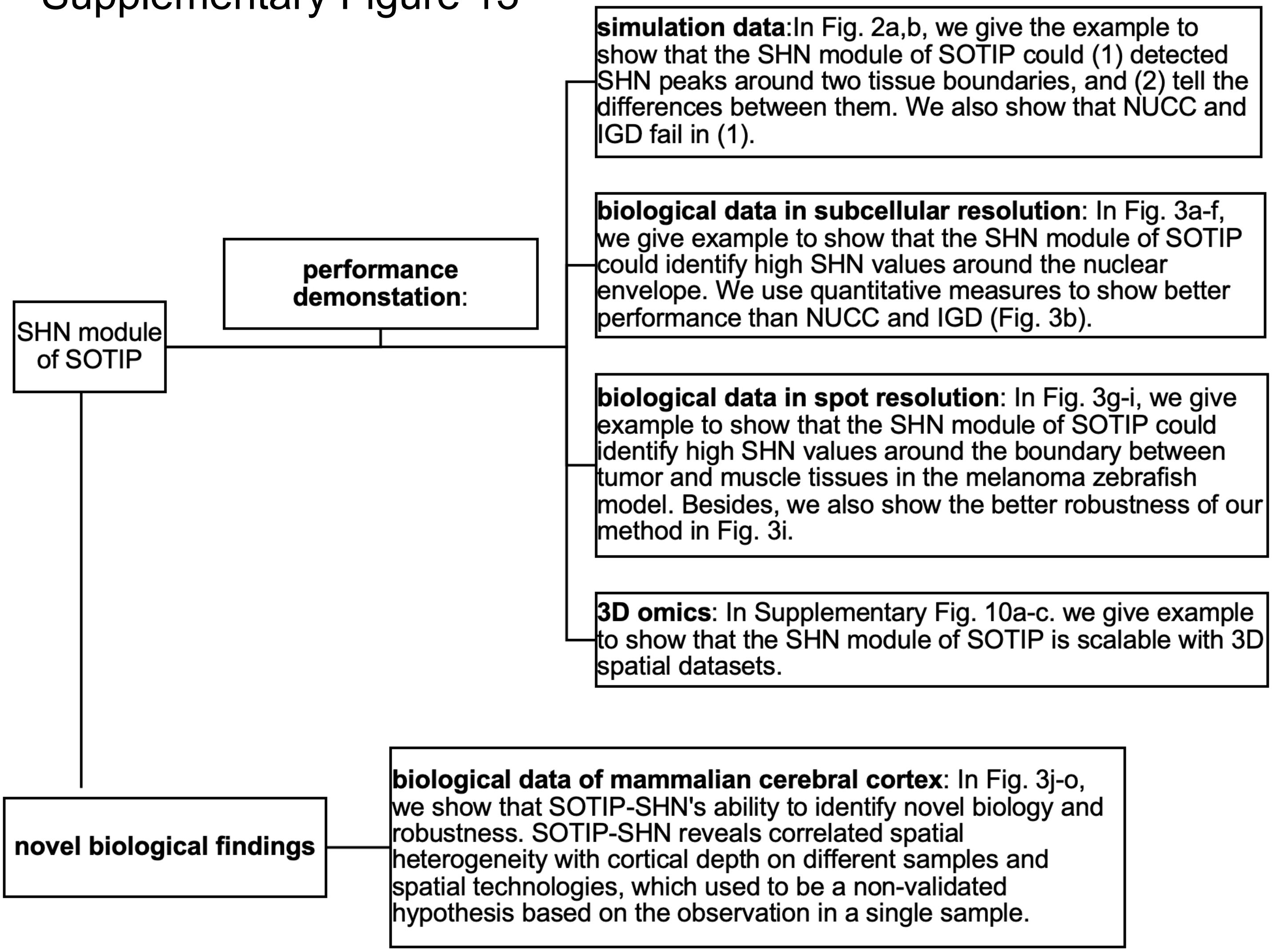

Supplementary Figure 13. Demonstration logic of SHN module

Supplementary Figure 14

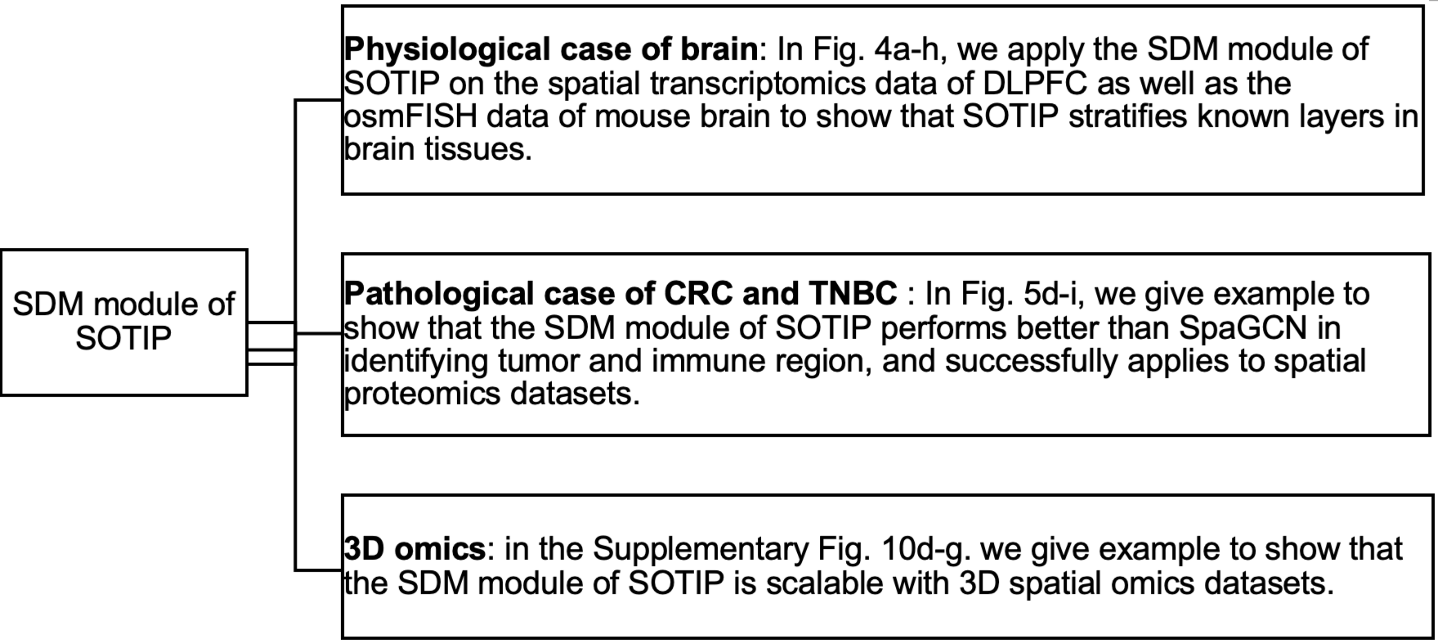

Supplementary Figure 14. Demonstration logic of SDM module

Supplementary Figure 15

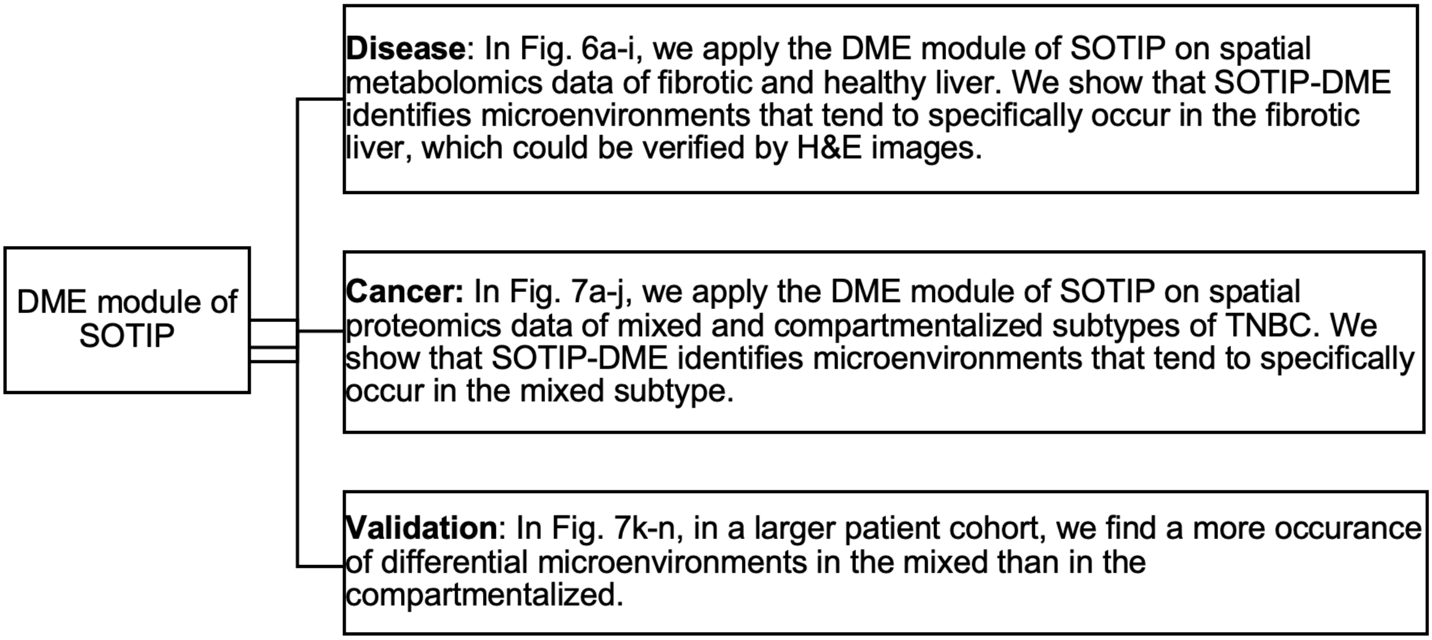

Supplementary Figure 15. Demonstration logic of DME module

Supplementary Tables

Supplementary Table 1: terms used in manuscript.

Term	Method	Task
**CCNR**[1-3]	Classical microenvironment (ME) representation and downstream analysis	ME representation
**HMRF**[4]	Probabilistic graphical model	Spatial domain identification
**BayesSpace**[5]	Probabilistic graphical model	Spatial domain identification
**SpaGCN**[6]	Graph convolution network	Spatial domain identification
**StLearn**[7]	Weighted clustering	Spatial domain identification
**SEDR**[8]	Graph convolution network	Spatial domain identification
**SpatialPCA**[9]	Probabilistic graphical model	Spatial domain identification
**STAGATE**[10]	Graph convolution network	Spatial domain identification
**Giotto**[11]	Probabilistic graphical model	Spatial domain identification
**NUCC**[12-14]	Number of unique cell clusters	Spatial heterogeneity quantification
**IGD**	Similar with SOTIP, but IGD use an isotropic ground distance to measure the dis-similarity between cell clusters (see Methods).	Spatial heterogeneity quantification
**CGMGD**	Connectivity guided minimum graph distance (see Methods)	Ground distance for MECN distance computing

Supplementary Table 2: Simulation data summary.

Name	Tool	Cell distribution	Cell composition	Spatial distribution	Purpose
Simulation 1	Splatter[15]	Two continuous groups and one discrete group, Supplementary Figure 1a	Supplementary Figure 1b	Randomly positioned these three cluster of cells as three sequential bands shaped tissue bulk on a two dimensional plane. Supplementary Figure 1c .	In order to verify the SHN methods could not only identify the existence of SHN peaks, but also distinguish the differences in SHN values.
Simulation 2	Splatter[15]	5 continuous groups (C1-C5), Supplementary Figure 1d	Supplementary Figure 1e	Regularly mixed these 5 clusters in 5 sequential bands on a two-dimensional plane (Fig. 1d right). In this data, each cell type occupies a major component in a band, and other cell types occupies minor components, and the order of major cell types along these tissue bands (R1 to R5) is agreed with the order of cell types along the gene expression manifold (C1 to C5) (Fig. 1d middle). Supplementary Figure 1f,g	In order to verify which graph construction method can be used to restore the structured manifold of microenvironments.
Simulation 3	Splatter[15]	3 discrete groups, Supplementary Figure 1h	Supplementary Figure 1i	Positioned the 3 cell clusters with two different order of sequential bands. Supplementary Figure 1j,k	To prove MEG could be used to identify microenvironments which differentiate between samples
Simulation 4	Splatter[15]	20 continuous groups,	Supplementary Figure 1m	None	Performance of CGMGD

		Supplementary Figure m			

Supplementary Table 3: Published datasets used in this study.

Species	Tissue	Source	Dimension	Protocol	Usage
Human	HeLa cell line	https://squidpy.readthedocs.io/en/stable/[16]	270,876 pixels<br>40 proteins<br>Total 13 cells.	4i	Spatial heterogeneity quantification
Mouse	Brain cortex	http://linnarssonlab.org/osmFISH[17]	5,328 cells<br>33 genes	osmFISH	Spatial heterogeneity quantification,<br>Spatial domain identification
Mouse	Brain cortex	https://github.com/CaiGroup/seqFISH-PLUS[18]	913 cells<br>10,000 genes	seqFISH+	Spatial heterogeneity quantification
Zebrafish	melanoma	GSE159709[19]	7,281 spots<br>20,589 genes	10X Visium	Spatial heterogeneity quantification
Human	Brain cortex	http://research.libd.org/spatialLIBD/[20]	151673:<br>3,639 spots<br>33,538 genes.<br>Total 12 samples	10X Visium	Spatial domain identification
Human	colorectal carcinoma (p16)	https://zenodo.org/record/3951613[21]	1023 cells<br>36 proteins	scMEP	Spatial domain identification
Human	colorectal carcinoma (p23)	https://zenodo.org/record/3951613[21]	1241 cells<br>36 proteins	scMEP	Spatial domain identification
Human	triple negative breast cancer (compartmentalized)	https://mibi-share.ionpath.com/[22]	Patient 4:<br>6643 cells<br>37 proteins	MIBI	Spatial domain identification.<br>Differential microenvironment analysis
Human	triple negative breast cancer (*compartmentalized*)	https://mibi-share.ionpath.com/[22]	Patient 9:<br>6139 cells<br>37 proteins	MIBI	Spatial domain identification.
Human	triple negative breast cancer (*mixed*)	https://mibi-share.ionpath.com/[22]	Patient 12:<br>6995 cells<br>37 proteins	MIBI	Spatial domain identification.<br>Differential microenvironment analysis

Mouse	Healthy liver	https://github.com/yuanzhiyuan/SEAM/tree/master/SEAM/data/raw_tar[23]	706 cells 244 metabolites	TOF-SIMS	Differential microenvironment analysis
Human	Fibrotic liver	https://github.com/yuanzhiyuan/SEAM/tree/master/SEAM/data/raw_tar[23]	906 cells 228 metabolites	TOF-SIMS	Differential microenvironment analysis
Mouse	Brain	https://janelia.figshare.com/articles/dataset/EASI-FISH_enabled_spatial_analysis_of_molecular_cell_types_in_the_lateral_hypothalamus/13749154[24]	36423 cells, 26 genes	EASI-FISH	Spatial domain identification.
Human	Breast cancer	https://doi.org/10.5281/zenodo.4752030[25]	81226 cells 24 proteins.	3D IMC	Spatial heterogeneity quantification, Spatial domain identification.

Supplementary Table 4 Algorithm Comparison (Functionality)

**Basic information**		**Functionality**							
Reference	journal	Spatial domain identification	Spatial pattern genes	Spatial heterogeneity quantification	Differential microenvironment analysis	Resolution enhancement	Spatial trajectory	Tissue hierarchy	Cell communication
stLearn[7]	bioRxiv	√	X	X	X	X	√	X	√
HMRF[4]	NBT	√	√	X	X	X	X	X	X
SpaGCN[6]	NM	√	√	X	X	X	X	X	X
SEDR[8]	bioRxiv	√	√	X	X	X	√	X	√
BayesSpace[5]	NBT	√	√	X	X	√	X	X	X
SCAN-IT[26]	BMVC	√	√	X	X	X	X	X	X
RESEPT[27]	bioRxiv	√	X	X	X	X	X	X	X
STAGATE[10]	bioRxiv	√	√	X	X	X	√	X	X
CCST[28]	Research Square	√	√	X	X	X	X	X	X
FICT[29]	Bioinformatics	√	X	X	X	X	X	X	X
Stereo[30]	Research Square	√	√	X	√	X	√	X	X
SpatialPCA[9]	bioRxiv	√	X	X	X	√	√	X	X
SC-MEB[31]	BIB	√	√	X	X	X	X	X	X
conST[32]	bioRxiv	√	√	X	X	X	√	X	√
SOTIP	Our manuscript	√	√	√	√	X	√	√	X

Supplementary Table 5: Algorithm Comparison (Method design)

Basic information		Method design				
Reference	journal	Interpretability	simulation	Unified	methodology	Independence of histology
stLearn[7]	bioRxiv	X	X	3	Weighted clustering	√
HMRF[4]	NBT	√	X	2	PGM	√
SpaGCN[6]	NM	X	X	1	DL	X
SEDR[8]	bioRxiv	X	X	2	DL	√
BayesSpace[5]	NBT	√	√	2	PGM	√
SCAN-IT[26]	BMVC	X	X	1	DL	√
RESEPT[27]	bioRxiv	X	X	1	DL	√
STAGATE[10]	bioRxiv	√	X	2	DL	√
CCST[28]	Research Square	X	X	1	DL	√
FICT[29]	Bioinformatics	X	√	1	PGM	√
Stereo[30]	Research Square	√	√	2	DL	√
SpatialPCA[9]	bioRxiv	√	√	3	PGM	√
SC-MEB[31]	BIB	X	√	1	PGM	√
conST[32]	bioRxiv	√	X	2	DL	√
SOTIP	Our manuscript	√	√	4	Graph	√

PGM: probabilistic graphical model

DL: deep learning

Supplementary Table 6: Algorithm Comparison (Code availability)

Basic information		Code availability		
Reference	journal	Software availability	Tutorial availability	Reproducible code availability
stLearn[7]	bioRxiv	Python	√	√
HMRF[4]	NBT	R	X	√
SpaGCN[6]	NM	Python	√	X
SEDR[8]	bioRxiv	Python	√	√
BayesSpace[5]	NBT	R	√	X
SCAN-IT[26]	BMVC	Python	X	√
RESEPT[27]	bioRxiv	Python	√	X
STAGATE[10]	bioRxiv	X	X	X
CCST[28]	Research Square	Python	X	X
FICT[29]	Bioinformatics	Python	X	X
Stereo[30]	Research Square	X	X	X
SpatialPCA[9]	bioRxiv	R	X	X
SC-MEB[31]	BIB	R	√	X
conST[32]	bioRxiv	X	X	X
SOTIP	Our manuscript	Python	√	√

Supplementary Table 7 Algorithm Comparison (Applicability)

Basic information		Applicability			
Reference	journal	Spatial transcriptomics	Spatial proteomics	Spatial metabolomics	3D
stLearn[7]	bioRxiv	10X Visium	X	X	X
HMRF[4]	NBT	seqFISH	X	X	X
SpaGCN[6]	NM	10X Visium, Slideseq, STARmap, MERFISH	X	X	X
SEDR[8]	bioRxiv	10X Visium, Stereoseq	X	X	X
BayesSpace[5]	NBT	10X Visium	X	X	X
SCAN-IT[26]	BMVC	osmFISH, seqFISH, 10X Visium, Slideseq	X	X	X
RESEPT[27]	bioRxiv	10X Visium	X	X	X
STAGATE[10]	bioRxiv	10X Visium, Slideseq, Stereoseq	X	X	Slideseq
CCST[28]	Research Square	10X Visium, Merfish, seqFISH	X	X	X
FICT[29]	Bioinformatics	seqFISH, MERFISH	X	X	X
Stereo[30]	Research Square	STARmap, 10X Visium	MIBI-TOF, CODEX	X	X
SpatialPCA[9]	bioRxiv	10X Visium, Slideseq	X	X	X
SC-MEB[31]	BIB	10X Visium	X	X	X
conST[32]	bioRxiv	10X Visium, MERFISH	X	X	X
SOTIP	Our manuscript	10X Visium, osmFISH, seqFISH, EASIFISH	4i, MIBI-TOF, scMEP	TOF-SIMS	EASIFISH 3D IMC

Supplementary Table 8. Datasets and biological systems

Module	Design of demonstration	Species	Tissue	Protocol	Detected molecule	Figure	Positive control
SHN	subcellular resolution	Human	HeLa cell line	4i	protein	Fig. 3a-f	The nuclear envelope should have highest spatial heterogeneity.
	cellular resolution; physiological case	Mouse	Brain cortex	osmFISH	mRNA	Fig. 3j-o	The spatial heterogeneity should have a gradient pattern towards deeper cortical layer.
		Mouse	Brain cortex	seqFISH+	mRNA		
	spot resolution; pathological case	Zebrafish	melanoma	10X Visium	mRNA	Fig. 3g-i	The tumor boundary should have highest spatial heterogeneity.
SDM	Physiological case; Brain	Human	Brain cortex	10X Visium	mRNA	Fig. 4a-d	Labeled region
		Mouse	Brain	osmFISH	mRNA	Fig. 4e-h	Labeled region
	pathological case; CRC	Human	colorectal carcinoma (p16)	scMEP	protein	Fig. 5d-e	Labeled region
		Human	colorectal carcinoma (p23)	scMEP	protein		
	pathological case; TNBC	Human	triple negative breast cancer (*compartme*	MIBI	protein	Fig. 5g-h	Labeled region

			ntalized, p4)				
		Human	triple negative breast cancer (*compartmentalized*, p9)	MIBI	protein		
DME	Normal tissue vs two subtypes of TNBC	Human	triple negative breast cancer (compartmentalized)	MIBI	protein	Fig. 7	The two subtypes should have different cell type spatial organization, and the difference should be consistent on other 30+ patients.
		Human	triple negative breast cancer (*mixed*)	MIBI	protein		
	Healthy liver vs Fibrotic liver	Mouse	Healthy liver	TOF-SIMS	metabolite	Fig. 6	There should be microenvironment difference between healthy and fibrotic liver. The H&E could be act as positive control.
		Human	Fibrotic liver	TOF-SIMS	metabolite		

Supplementary Table 9. Terms

Term	Explanation
ISS	In Situ Sequencing
smFISH	single-molecule Fluorescence In Situ Hybridization
MERFISH	Multiplexed Error-Robust Fluorescence In Situ Hybridization
seqFISH	Sequential Fluorescence In Situ Hybridization
ST	Spatial Transcriptomics
HDST	High-Definition Spatial Transcriptomics
4i	iterative indirect immunofluorescence imaging
CODEX	CO-DEtection by indeXing
MIBI-TOF	Multiplexed Ion Beam Imaging by Time-Of-Flight
IMC	Imaging Mass Cytometry
AFADESI-MSI	AirFlow-Assisted Desorption ElectroSpray Ionization Mass Spectrometry Imaging
SEAM	Spatial single nuclEAr Metabolomics
ME	MicroEnvironment
KNN graph	We referred to reference #32
CCNR	Classical Cell Neighborhood Representation, and we referred to Supplementary Table 1 and reference #29~31
NUCC	Number of Unique Cell Clusters, and we referred to Supplementary Table 1 and reference #16,33,35
BayesSpace	It is the name of an existing algorithm, and we referred to Supplementary Table 1 and reference #37
HMRF	Hidden-Markov Random Field
SpaGCN	It is the name of an existing algorithm, and we referred to Supplementary Table 1 and reference #38
GCN	Graph Convolutional Network
StLearn	It is the name of an existing algorithm, and we referred to Supplementary Table 1 and reference #39
scRNA-seq	single-cell RNA sequencing
SHN	Spatial HeterogeNeity
SDM	Spatial DoMain
DME	Differential MicroEnvironment
MECN	Molecular-Expression-aware Cellular Neighborhood
MEG	MECN Graph
TNBC	Triple Negative Breast Cancer
MKT	Highly specific MECNs identified by DME
SIMS	Secondary Ion Mass Spectrometry
IGD	An algorithm for quantifying SHN, we referred to Supplementary Table 1, where it is further linked to Methods section for detailed explanation.
UMAP	A widely used manifold learning method, we referred to Reference #57
PHATE	A widely used manifold learning method, we referred to Reference #58
DNA	We did not define it since it has been widely known as Deoxyribonucleic acid.

Squidpy	A python package for processing spatial omics data, we referred to Reference #61
HeLa	We did not define it since it has been widely known as a class of cell line.
AUC	Area Under Curve
PCNA	Proliferating Cell Nuclear Antigen
RPS6	Ribosomal Protein S6
ER	Endoplasmic Reticulum
BRAFV600E	A melanoma zebrafish model, we referred to Reference #63
Leiden	An existing clustering algorithm, we referred to Reference #64
Pearson's r	Pearson correlation coefficient
BICCN	BRAIN Initiative Cell Census Network, and we referred to Reference #67
osmFISH	cyclic-ouroboros single molecule fluorescence in situ hybridization, and we referred to Reference #52
Spearman's ρ	Spearman's rank correlation coefficients
seqFISH+	evolution of sequential fluorescence in situ hybridization, and we referred to Reference #17
FOV	Field Of View
SpatialLIBD	A spatial transcriptomics dataset, we referred to Reference 54
DLPFC	DorsoLateral PreFrontal Cortex
k-means	A widely used clustering algorithm
Louvain	A widely used clustering algorithm, we referred to Reference #64
SC3	A widely used clustering algorithm, we referred to Reference #68
Giotto	A spatial omics data processing package, we referred to Reference #69
SEDR	An existing spatial domain identification algorithm, we referred to Reference #40
STAGATE	An existing spatial domain identification algorithm, we referred to Reference #41
SpatialPCA	An existing spatial domain identification algorithm, we referred to Reference #42
ARI	Adjusted Rand Index
MET	MicroEnvironment Trajectory
PAGA	One of the most widely used trajectory inference method, we referred to Reference #70
scMEP	A spatial proteomics technique, we referred to Reference #55
CRC	ColoRectal Carcinoma
FDR	False-Discovery Rate
DMA	Differential Microenvironment Analysis
EH plot	"entropy-of-ME-cluster (EMC)" versus "spatial heterogeneity (SHN)" plot, and we also referred to Methods for detailed explanation.
EMC	entropy-of-ME-cluster, and we also referred to Methods for detailed explanation.
NPC	Non-hepatic Parenchymal Cells
TILs	Tumor-infiltrating lymphocytes

EASI-FISH	3D spatial transcriptomics, we also referred to Reference #53
3D IMC	3D spatial proteomics, we also referred to Reference #91
WB ratio	Within-cluster-Between-cluster ratio
EMD	Earth mover’s distance, and we also referred to Reference #96
CGMGD	Connectivity Guided Minimum Graph Distance, and we also referred to “Connectivity guided minimum graph distance (CGMGD)” in Methods section for detail explanation.
PGEV	Pairwise Gene Expression Variation, where we also explained how to compute it.
NCME	Normalized count of MECNs, where we also explained how to compute it.
t-SNE	A famous manifold learning algorithm, and we referred to Reference #100
DPT	One of the most widely used trajectory inference algorithm, and we referred to Reference #104
BCM	Binary Connectivity Matrix
UDM	UMAP Distance Matrix
CGG	Connectivity Guided cluster Graph
ForceAtlas	A manifold learning algorithm, and we referred to Reference #105
Splatter	A single cell simulation tool, and we referred to Reference #106
MELD	An algorithm to compute the relative likelihood of observing samples in different conditions. We referred to Reference #46
BH	Benjamini–Hochberg

Supplementary Table 10. Additional comparison

	SOTIP	Squidpy	RESEPT	BayesSpace	Cell2Location
Method principle	Optimal transport	Integrated computational framework	Deep learning	Probabilistic graphical models	Probabilistic graphical models
Spatial heterogeneity quantification	√				
Spatial domain identification	√		√	√	
Differential microenvironment analysis	√				
neighborhood enrichment analysis		√			
co-occurrence analysis		√			
interaction analysis		√			
autocorrelation analysis		√			
spatially variable gene analysis		√			
Image processing		√			
Enable running on multiple samples	√			√	
Resolution enhancement				√	
Cell type decomposition					√

Supplementary Notes

Supplementary Note 1. Parameter discussion.

The parameter setting is consistent across this manuscript. There are three parameters for user to choose according to their applications.

The first one is the resolution of Leiden clustering. This parameter controls the initial clustering resolution before MECN definition. Since SOTIP could incorporate the distances between cell clusters into MECN representation, when the resolution is set too high (over-clustering), the algorithm can still remedy the error with its characteristics, but when the resolution is set too low (under-clustering), the reliability of constructed MEG could be compromised. As a result, users should rather set this parameter too high than set it too low. Across our manuscript, for datasets with true number of cell clusters, we provided a function for searching the modest resolution, and for datasets without true number of cell clusters ,we set the Leiden resolution to 2. We also tested SOTIP's SHN quantification performance with different clustering resolutions on osmFISH (Supplementary Fig. 2a,b), seqFISH+ (Supplementary Fig. 2e,f), and Visium datasets (Fig. 2i, Supplementary Fig. 2i,j), the results showed that SOTIP performed better than other methods, even with inappropriate setting of parameters (e.g. over clustering).

The second one is how to define MECNs. We don't use the Voronoi tessellation graph because it is reported that cells resided in microenvironment could perform interplay within broader scales than nearest cells. Since different tissues have different ranges of cell-cell interactions (for example the neuron cells have wider spatial range of interplays because of the presence of axons), using the KNN spatial graph would allow users to freely adjust the scale of MECN according to applications or tissue prior. Across the manuscript, we used kNN search for each cell to define k nearest cells/spots as a microenvironment. For all datasets in our manuscript, k is set to 10 consistently, except for the SHN quantification in cerebral cortex FISH dataset for evaluating SOTIP's robustness to MECN size. SOTIP's SHN quantification performance is also validated to be better than other methods, with various choices of MECN sizes (Supplementary Fig. 2a,b,e,f).

The third one is the number of clusters in SDM identification. Following the widely used methodology[5,6], we set the number of clusters to the true number of clusters. In the real applications, where the true number of clusters is not known a prior, SOTIP can provides users with a multi-level tissue partitioning hierarchy, from which tissue regions with different resolutions can be investigated.

Supplementary Note 2. Further explanations on SHN.

SOTIP's SHN (Spatial heterogeneity quantification) module is used to quantify the gene expression variance of cells within a spatial neighborhood. Since each cell has a spatial neighborhood, containing a number of cells, the output of SHN is a scalar value per cell. If a cell's associated spatial neighborhood contains cells with very different gene expression profiles, the SHN value of this cell is high, meaning spatially more heterogeneous. The only control method of SHN is NUCC (IGD mentioned in the original manuscript is not a previous method, but a variant of SOTIP), which simply regards the number of unique cell clusters within each neighborhood as spatial heterogeneity. SOTIP-SHN considers the gene expression variation within neighborhood, and NUCC considers the cell cluster variation within neighborhood. That being said, SOTIP puts the cell relationships into a gene expression space so that distance between cells could be computed by distance metrics between gene expression profiles, but NUCC puts the cell relationships into a one-hot cell type representation space so that the relative distances between cells on the gene expression manifold are missed. So, SOTIP-SHN has higher resolution than NUCC. Because SOTIP-SHN considers gene expression relationships between cells, it is not restricted by the accuracy of cell clustering, but the reliability of NUCC could be easily influenced by the accuracy of cell clustering. SOTIP-SHN is especially useful when continuity pronounced in gene expression space (as demonstrated in Figure 2a,b), or when it is difficult to choose a cell clustering resolution (as demonstrated in Figure 3i and Supplementary Figure 2a-d).

Supplementary Note 3. Further explanations on DME.

SOTIP's DME (differential microenvironment analysis) module is used to identify those microenvironments which differentiate between two spatial omics tissue sections. Since there is currently no other methods for spatial omics data that can do the same thing as SOTIP-DME, we did not compare SOTIP-DME with other spatial methods. However, there exist non-spatial methods that can identify cell states that can differentiate between two samples, for example, MELD[33], Milo[34], and CNA[35]. The main difference between SOTIP-DME and these methods are that SOTIP-DME additionally utilizes the spatial information. Regarding this, SOTIP-DME would perform better than these single cell non-spatial methods when two samples share similar cell state composition but have different cell spatial organization. The TNBC case in Figure 7 has demonstrated this strength.

Supplementary Note 4. Comparison with Squidpy.

Squidpy integrates neighborhood enrichment analysis, co-occurrence analysis, interaction analysis, autocorrelation analysis, and spatially variable gene analysis, as well as image processing tools for histological image analysis. We compared SOTIP with Squidpy in Supplementary Table 10. Specifically, SOTIP's key principle is optimal transport, while Squidpy integrated a large number of existing algorithms into a unified framework. SOTIP provides functions such as Spatial heterogeneity quantification (SHN), Spatial domain identification (SDM), and Differential microenvironment analysis (DME). While Squidpy provides functions such as neighborhood enrichment analysis, co-occurrence analysis, interaction analysis, autocorrelation analysis, spatially variable gene analysis, and image processing.

Supplementary Note 5. Comparison with RESEPT.

RESEPT is a supervised deep learning method for spatial domain identification. According to RESEPT's manuscript (https://doi.org/10.1101/2021.07.08.451210) line 33~38, RESEPT firstly learns a three-dimensional embedding using a graph autoencoder from the spatial transcriptomics data. The embedding is then visualized by mapping as color channels in an RGB image and segmented with a supervised convolutional neural network model. The comparison between RESEPT and SOTIP is in Supplementary Table 10. The common part between RESEPT and SOTIP is the spatial domain identification module. The different part between them is two folds:

(1) For spatial domain identification (SDM), SOTIP is an unsupervised method which does not require any external datasets for training, while RESEPT requires external datasets for training before spatial domain inference.
(2) SOTIP is a versatile method, which can perform multiple tasks, in which SDM is one of them.

Supplementary Note 6. Comparison of SOTIP-SDM with other related methods.

For clustering procedure, SOTIP followed a hierarchical merging procedure, and the intermediate clustering result could be maintained and evaluated in just one-time running. While other three methods do not enjoy this benefit due to their different principles. Specifically, SpaGCN needs to initialize the cluster labels with predefined number of clusters, then applying the iterative clustering procedure to stochastically optimize parameters and cluster labels. As a probabilistic graphical model, BayesSpace also needs predefined number of clusters to formulize the model. It regarded the labels as hidden variable, then building a fully Bayesian model with Gaussian as conditional distribution and Markov random field as smooth prior. As to stLearn, it adopts either k-means or Louvain to perform clustering after generating the normalized graph adjacency matrix. Because the true number of clusters is not accessible, which is the common situation in real applications, all these three methods need to constantly re-run entire program to test different number clusters or resolution parameters.

For the extra information usage, as with BayesSpace, SOTIP does not require incorporate histological information (e.g., H&E) to obtain better performance than SpaGCN and stLearn. In the common situation, producing spatial omics data together with paired histological image needs additional efforts on experimental design and finer operations, so that many mainstream spatial omics researches would not actually provide paired or vertically adjacent histological images[13,18,21,22,36-38]. What's more, the discontinuity of adjacent sections and the uncontrollable noise further complicate the utility of histological information.

Supplementary Note 7. SOTIP delineates microenvironment trajectory of human cerebral cortex.

Inspired by single cell trajectory inference, SOTIP provided a microenvironment trajectory (MET) inference module. The MECN graph built by SOTIP effectively represented the relationships among microenvironments. We applied PAGA[39] to delineate the topological structure of MECNs, with SpatialLIBD[20] human cortex 10X Visium datasets. We used this dataset to demonstrate MET because this dataset provides the cortical layer annotation and the cerebral cortex naturally displays an ordinal spatial pattern as ground truth. We adopted SOTIP to perform MET inference on all 12 samples, the results showed high consistency between inferred trajectory and the cortex layer order (Supplementary Fig. 3).

Supplementary Note 8. Literature validations for MECN 24 found in Figure 7.

Above analysis implied that MECN cluster 24 (CD8 T, macrophage, and Keratin+ tumor) may drive the difference between *compartmentalized* and *mixed* subtypes of TNBC. Tumor-infiltrating lymphocytes (TILs) have aroused wide interest because of its prognostic significance[40]. As an important part of TILs, CD8 T cells have been proved to be associated with lots of immune checkpoint molecules as well as the survival rate of patients[41,42]. However, a pooled analysis[43] containing 3,771 patients showed that increased TILs were not always a positive prognostic factor for breast cancer patients' survival. How TILs related to the prognosis of patients differed in breast cancer subtypes. For example, high-level TILs contradicted with the survival benefit in patients of luminal–HER2-negative breast cancer. On the other hand, a recent study revealed that macrophages played a role in excluding CD8 T cells from reaching the tumor cells, thus causing a poor clinical outcome[44]. Combining these studies, an assumption came up that TILs should help to fight against tumor but it may be hindered by other cells in the microenvironment. Once the hindering happened, it would indicate a condition where immunocompetent hosts failed to defend against the antigenic tumor. For example, the mixture of CD8 T cells and macrophages could be regarded as a malignant signal indicating bad survival chance. This assumption was supported by our finding that MECN cluster 24 specifically occurred more in the *mixed* group (reported to be more malignancy and output worse survival[22]) than in the *compartmentalized* group across the patient cohort. Altogether, by fully integrating the spatial information and quantitatively measuring the microenvironment difference between TNBC samples, our analysis demonstrated a solid case to show the power of our ME-based computational framework. Our findings suggested a potential theory to explain why compartmentalized-pattern TNBC patients would response better than the mix-pattern ones and exhibited the potential of MECN as a powerful clinical indicator in a biologically meaningful way.

Supplementary Note 9. SOTIP is scalable with 3D spatial omics datasets.

Since there are currently no method for SDM identification in 3D spatial datasets, we next applied SOTIP on two recently published 3D spatial omics datasets, i.e., EASI-FISH[24] and 3D IMC[25]. EASI-FISH[24] dataset contains spatial transcriptomics measurement of 36,422 single cells in thick brain slice of lateral hypothalamus region. The results showed an agreement between the anatomical annotation and the low-dimensional UMAP embedding computed with MEG (Supplementary Fig. 9a). The within-cluster-between-cluster ratio (WB ratio) also showed significant agreement between the MEG and anatomical annotation (Supplementary Fig. 9b).

On 3D IMC[25] dataset, we applied SOTIP to perform spatial heterogeneity quantification and spatial domain identification on a Her2 breast carcinoma model, processed by 3D IMC. The results are shown in Supplementary Figure 10. Specifically, supplementary Figure 10a shows the spatial heterogeneity results in 3D. For visualization, supplementary Figure 10b,c shows in silico sliced sections along z-axis. These results shows consistently higher spatial heterogeneity around the tumor-stroma boundary. Supplementary Figure 10d shows the spatial domain identification results in 3D. For visualization, supplementary Figure 10e,f shows in silico sliced sections along z-axis. These results shows SOTIP successfully stratifies different tissue regions (i.e. tumor and stroma region), consistent with the marker enrichment (Supplementary Fig. 10g).

Supplementary Note 10. Computational resource.

Across the manuscript, all the results are run on a machine with following computational resources.

Item	Information
**Operation System**	CentOS Linux release 7.9
**Number of CPUs**	16
**Number of GPUs**	0
**Memory Size**	31.1G

This means that the required computational resources are recommended to successfully run our analyses. Machines with lower computational resources may also be enough depending on analysis. We listed the expected time of different analyses as follows:

Analysis	Number of spots/cells	Recommend multiprocessing	Expected time
**SHN**	10^2	No	<1min
**SHN**	10^3	No	<1min
**SHN**	10^4	No	1min~5min
**SDM**	10^2	Yes	<1min
**SDM**	10^3	Yes	1min~5min
**SDM**	10^4	Yes	5min~10min
**DME**	10^2	Yes	<1min
**DME**	10^3	Yes	1min~5min
**DME**	10^4	Yes	5min~10min

References

1 Rovira-Clave, X. *et al.* Subcellular localization of biomolecules and drug distribution by high-definition ion beam imaging. *Nat Commun* **12**, 4628, doi:10.1038/s41467-021-24822-1 (2021).

2 Bhate, S. S., Barlow, G. L., Schürch, C. M. & Nolan, G. P. Tissue schematics map the specialization of immune tissue motifs and their appropriation by tumors. *Cell Syst*, doi:10.1016/j.cels.2021.09.012 (2021).

3 Schürch, C. M. *et al.* Coordinated Cellular Neighborhoods Orchestrate Antitumoral Immunity at the Colorectal Cancer Invasive Front. *Cell*, doi:10.1016/j.cell.2020.07.005 (2020).

4 Zhu, Q., Shah, S., Dries, R., Cai, L. & Yuan, G. C. Identification of spatially associated subpopulations by combining scRNAseq and sequential fluorescence in situ hybridization data. *Nat Biotechnol* **36**, 1183-+, doi:10.1038/nbt.4260 (2018).

5 Zhao, E. *et al.* Spatial transcriptomics at subspot resolution with BayesSpace. *Nat Biotechnol*, doi:10.1038/s41587-021-00935-2 (2021).

6 Hu, J. *et al.* SpaGCN: Integrating gene expression, spatial location and histology to identify spatial domains and spatially variable genes by graph convolutional network. *Nat Methods* **18**, 1342-1351, doi:10.1038/s41592-021-01255-8 (2021).

7 Pham, D. *et al.* stLearn: integrating spatial location, tissue morphology and gene expression to find cell types, cell-cell interactions and spatial trajectories within undissociated tissues. *bioRxiv* (2020).

8 Fu, H. *et al.* Unsupervised Spatial Embedded Deep Representation of Spatial Transcriptomics. *bioarxiv*, doi:10.1101/2021.06.15.448542 (2021).

9 Shang, L. & Zhou, X. Spatially Aware Dimension Reduction for Spatial Transcriptomics. doi:10.1101/2022.01.19.476966 (2022).

10 Dong, K. & Zhang, S. Deciphering spatial domains from spatially resolved transcriptomics with adaptive graph attention auto-encoder. doi:10.1101/2021.08.21.457240 (2021).

11 Dries, R. *et al.* Giotto: a toolbox for integrative analysis and visualization of spatial expression data. *Genome Biol* **22**, 78, doi:10.1186/s13059-021-02286-2 (2021).

12 Chen, H. *et al.* Dissecting Mammalian Spermatogenesis Using Spatial Transcriptomics. *bioRxiv*, doi:10.1101/2020.10.17.343335 (2020).

13 Zhang, M. *et al.* Spatially resolved cell atlas of the mouse primary motor cortex by MERFISH. *Nature* **598**, 137-143, doi:10.1038/s41586-021-03705-x (2021).

14 Moffitt, J. R. *et al.* Molecular, spatial, and functional single-cell profiling of the hypothalamic preoptic region. *Science* **362**, doi:10.1126/science.aau5324 (2018).

15 Zappia, L., Phipson, B. & Oshlack, A. Splatter: simulation of single-cell RNA sequencing data. *Genome Biol* **18**, 174, doi:10.1186/s13059-017-1305-0 (2017).

16 Gut, G., Herrmann, M. D. & Pelkmans, L. Multiplexed protein maps link subcellular organization to cellular states. *Science* **361**, doi:10.1126/science.aar7042 (2018).

17 Codeluppi, S. *et al.* Spatial organization of the somatosensory cortex revealed by osmFISH. *Nat. Methods* **15**, 932-+, doi:10.1038/s41592-018-0175-z (2018).

18 Eng, C. L. *et al.* Transcriptome-scale super-resolved imaging in tissues by RNA seqFISH. *Nature* **568**, 235-239, doi:10.1038/s41586-019-1049-y (2019).

19 Hunter, M. V., Moncada, R., Weiss, J. M., Yanai, I. & White, R. M. Spatially resolved

transcriptomics reveals the architecture of the tumor-microenvironment interface. *Nat Commun* **12**, 6278, doi:10.1038/s41467-021-26614-z (2021).

20 Maynard, K. R. *et al.* Transcriptome-scale spatial gene expression in the human dorsolateral prefrontal cortex. *Nat Neurosci* **24**, 425-436, doi:10.1038/s41593-020-00787-0 (2021).

21 Hartmann, F. J. *et al.* Single-cell metabolic profiling of human cytotoxic T cells. *Nat Biotechnol*, doi:10.1038/s41587-020-0651-8 (2020).

22 Keren, L. *et al.* A Structured Tumor-Immune Microenvironment in Triple Negative Breast Cancer Revealed by Multiplexed Ion Beam Imaging. *Cell* **174**, 1373-+, doi:10.1016/j.cell.2018.08.039 (2018).

23 Yuan, Z. *et al.* SEAM is a spatial single nuclear metabolomics method for dissecting tissue microenvironment. *Nat. Methods* **18**, 1223-1232, doi:10.1038/s41592-021-01276-3 (2021).

24 Wang, Y. *et al.* EASI-FISH for thick tissue defines lateral hypothalamus spatio-molecular organization. *Cell* **184**, 6361-6377 e6324, doi:10.1016/j.cell.2021.11.024 (2021).

25 Kuett, L. *et al.* Three-dimensional imaging mass cytometry for highly multiplexed molecular and cellular mapping of tissues and the tumor microenvironment. *Nature Cancer* **3**, 122-133, doi:10.1038/s43018-021-00301-w (2021).

26 Zixuan Cang, X. N., Annika Nie, Min Xu and Jing Zhang. SCAN-IT: Domain segmentation of spatial transcriptomics images by graph neural network. (2021).

27 Chang, Y. *et al.* Define and visualize pathological architectures of human tissues from spatially resolved transcriptomics using deep learning. doi:10.1101/2021.07.08.451210 (2021).

28 Li, J., Chen, S., Pan, X., Yuan, Y. & Shen, H.-b. CCST: Cell clustering for spatial transcriptomics data with graph neural network. doi:10.21203/rs.3.rs-990495/v1 (2021).

29 Teng, H., Yuan, Y. & Bar-Joseph, Z. Clustering Spatial Transcriptomics Data. *Bioinformatics*, doi:10.1093/bioinformatics/btab704 (2021).

30 Yang, M. *et al.* Position-informed contrastive learning for spatially resolved omics deciphers hierarchical tissue structure at both cellular and niche levels. doi:10.21203/rs.3.rs-1067780/v1 (2022).

31 Yang, Y. *et al.* SC-MEB: spatial clustering with hidden Markov random field using empirical Bayes. *Brief Bioinform* **23**, doi:10.1093/bib/bbab466 (2022).

32 Zong, Y. *et al.* conST: an interpretable multi-modal contrastive learning framework for spatial transcriptomics. doi:10.1101/2022.01.14.476408 (2022).

33 Burkhardt, D. B. *et al.* Quantifying the effect of experimental perturbations at single-cell resolution. *Nat Biotechnol*, doi:10.1038/s41587-020-00803-5 (2021).

34 Dann, E., Henderson, N. C., Teichmann, S. A., Morgan, M. D. & Marioni, J. C. Differential abundance testing on single-cell data using k-nearest neighbor graphs. *Nat Biotechnol*, doi:10.1038/s41587-021-01033-z (2021).

35 Reshef, Y. A. *et al.* Co-varying neighborhood analysis identifies cell populations associated with phenotypes of interest from single-cell transcriptomics. *Nat Biotechnol*, doi:10.1038/s41587-021-01066-4 (2021).

36 Stickels, R. R. *et al.* Highly sensitive spatial transcriptomics at near-cellular resolution with Slide-seqV2. *Nat Biotechnol*, doi:10.1038/s41587-020-0739-1 (2020).

37 Rodriques, S. G. *et al.* Slide-seq: A scalable technology for measuring genome-wide expression at high spatial resolution. *Science* **363**, 1463-+, doi:10.1126/science.aaw1219 (2019).

38 Goltsev, Y. *et al.* Deep Profiling of Mouse Splenic Architecture with CODEX Multiplexed Imaging. *Cell* **174**, 968-981 e915, doi:10.1016/j.cell.2018.07.010 (2018).

39 Wolf, F. A. *et al.* PAGA: graph abstraction reconciles clustering with trajectory inference through a topology preserving map of single cells. *Genome Biol* **20**, 59, doi:10.1186/s13059-019-1663-x (2019).

40 Yu, P. & Fu, Y.-X. Tumor-infiltrating T lymphocytes: friends or foes? *Laboratory investigation* **86**, 231-245 (2006).

41 Ikeda, Y. *et al.* Clinical significance of T cell clonality and expression levels of immune-related genes in endometrial cancer. *Oncology reports* **37**, 2603-2610 (2017).

42 Li, F. *et al.* The association between CD8+ tumor-infiltrating lymphocytes and the clinical outcome of cancer immunotherapy: A systematic review and meta-analysis. *EClinicalMedicine* **41**, 101134 (2021).

43 Denkert, C. *et al.* Tumour-infiltrating lymphocytes and prognosis in different subtypes of breast cancer: a pooled analysis of 3771 patients treated with neoadjuvant therapy. *The lancet oncology* **19**, 40-50 (2018).

44 Peranzoni, E. *et al.* Macrophages impede CD8 T cells from reaching tumor cells and limit the efficacy of anti–PD-1 treatment. *Proceedings of the National Academy of Sciences* **115**, E4041-E4050 (2018).
