# Supplementary material for: SOTIP is a versatile method for microenvironment modeling with spatial omics data
Source: Nat Commun. 2022 Nov 28;13:7330. doi: 10.1038/s41467-022-34867-5 (PMC9705407; doi:10.1038/s41467-022-34867-5)
Supplement: Supplementary file 3 — Editorial Assessment Report [file 41467_2022_34867_MOESM3_ESM.pdf]

nature portfolio

GUIDED OPEN ACCESS

nature methods | nature communications | communications biology

Contents of this report

1. **Manuscript details**: overview of your manuscript and the editorial team.
2. **Review synthesis**: summary of the reviewer reports provided by the editors.
3. **Editorial recommendation**: personalized evaluation and recommendation from all 3 journals.
4. **Annotated reviewer comments**: the referee reports with comments from the editors.
5. **Open research evaluation**: advice for adhering to best reproducibility practices.

About the editorial process

Because you selected the **Nature Portfolio Guided Open Access** option, your manuscript was assessed for suitability in three of our titles publishing high-quality work across the spectrum of methods research: ***Nature Methods***, ***Nature Communications***, and ***Communications Biology***. More information about Guided Open Access can be found here.

Collaborative editorial assessment

Your editorial team discussed the manuscript to determine its suitability for the Nature Portfolio Guided OA pilot. Our assessment of your manuscript takes into account several factors, including whether the work meets the **technical standard** of the Nature Portfolio and whether the findings are of **immediate significance** to the readership of at least one of the participating journals in the Nature Portfolio Guided Open Access methods cluster.

Peer review

Experts were asked to evaluate the following aspects of your manuscript:

- **Novelty** in comparison to prior publications;
- **Likely audience** of researchers in terms of broad fields of study and size;
- **Potential impact** of the study on the immediate or wider research field;
- **Evidence** for the claims and whether additional experiments or analyses could feasibly strengthen the evidence;
- **Methodological detail** and whether the manuscript is reproducible as written;
- Appropriateness of the **literature review**.

Editorial evaluation of reviews

Your editorial team discussed the potential suitability of your manuscript for each of the participating journals. They then discussed the revisions necessary in order for the work to be published, keeping each journal's specific editorial criteria in mind.

Journals in the Nature portfolio will support authors wishing to transfer their reviews and (where reviewers agree) the reviewers' identities to journals outside of Springer Nature.
If you have any questions about review portability, please contact our editorial office at guidedoa@nature.com.

Manuscript details

Tracking number	Submission date	Decision date	Peer review type
GUIDEDOA-21-00434	Mar 3, 2022	Apr 21, 2022	Single-blind

Manuscript title

SOTIP is a Versatile Method for Microenvironment Modelling with Spatial Omics Data

Author details

Michael Zhang

Affiliation: University of Texas at Dallas

Editorial assessment team

Primary editor	**George Inglis**<br>Home journal: ***Communications Biology***<br>ORCID: **0000-0002-9069-5242**<br>Email: **george.inglis@us.nature.com**
Other editors consulted	**Lin Tang**<br>Home journal: ***Nature Methods***<br>ORCID: **0000-0002-6050-0424**<br><br>**Ilse Ariadna Valtierra Gutierrez**<br>Home journal: ***Nature Communications***<br>ORCID: **0000-0003-4128-5914**
About your primary editor	George received his PhD in Genetics and Molecular Biology from Emory University, where he studied mouse models of voltage-gated sodium channel dysfunction and epilepsy. He also has research experience in epigenomics and *in vitro* models of neuronal development. George joined the editorial team of *Communications Biology* in September 2020 and is based in the New York office.

Editorial assessment and review synthesis

**Editor's summary and assessment**	Here, the authors present SOTIP, a spatial -omics method that can integrate several analyses, including heterogeneity quantification (SHN), domain quantification (SDM), and differential microenvironment analysis (DME), among other tasks. They first use SOTIP on 3 simulated scRNA-seq datasets, showing that only SOTIP (and not alternative methods) can correctly resolve spatial boundaries (e.g. SHN) and maintain spatial distribution across various embedding algorithms (like UMAP, etc). They also demonstrate that SOTIP can identify differential cluster boundaries from scRNA-seq data with identical cell composition (but different spatial information). SOTIP is validated on several existing datasets from HeLa cells, zebrafish melanoma, mouse cortex, and human brain (among others), demonstrating new insight that SOTIP can provide over existing tools, altogether concluding that SOTIP could provide new angles to analyze and utilize spatial -omic data.<br><br>The editors jointly decided to send this manuscript out to review based on the strength of benchmarking, extensive case studies of SOTIP applications, and potential new features offered through this method. However, there was some concern about the overarching conceptual novelty, given the existence of methods to probe the various dimensions of spatial -omics datasets, including spatial heterogeneity and domain quantification.
**Editorial synthesis of reviewer reports**	The reviewers largely find the rationale behind SOTIP to be timely, and that the method could be a useful tool for the field. Reviewers #2-4, however, also raise serious concerns about the readability and technical advance, and request further benchmarking and simulations of how SOTIP would perform in alternate scenarios.<br><br>In light of this feedback, *Nature Communications* would be interested in a revised manuscript that addresses all the reviewer concerns in full. In addition to all the additional benchmarking and simulations required by the Reviewers, we would expect your revision to explore the possibility of analysing multiple samples in order to address the comments from Reviewer #2.<br><br>Similarly, *Communications Biology* would be interested in a revised manuscript that benchmarks SOTIP to at least one alternative method posed by Reviewer #2, incorporates at least one of the additional simulations noted by Reviewers #3-4, and includes proofreading for clarity and readability (with particular attention to the points from Reviewers #2-3).

Editorial recommendation

Nature Methods<br><br>Revision not invited	Neither the conceptual advance nor advance in performance demonstrated is sufficient for publication in *Nature Methods*.
Nature Communications<br><br>Major revisions with extension of the work	*Nature Communications* would be interested in a revised manuscript that addresses all the reviewer concerns in full. In addition to all the additional benchmarking and simulations required by the Reviewers, we would expect your revision to explore the possibility of analysing multiple samples in order to address the comments from Reviewer #2.
Communications Biology<br><br>Major revisions	*Communications Biology* would be interested in a revised manuscript that benchmarks SOTIP to at least one alternative method posed by Reviewer #2, incorporates at least one of the additional simulations noted by Reviewers #3-4, and includes proofreading for clarity and readability (with particular attention to the points from Reviewers #2-3).

Next steps

**Editorial recommendation 1:**	Our top recommendation is to revise and resubmit your manuscript to *Nature Communications*. We feel the additional required revisions could be completed within a 6-month time frame.
**Editorial recommendation 2:**	You may also choose to revise and resubmit your manuscript to *Communications Biology*. This option may be preferred if not all requested revisions are feasible to achieve at this time.
**Note:**	As stated on the previous page, *Nature Methods* is not inviting a revision at this time. Please keep in mind that the journal will not be able to consider any appeals of their decision through Guided Open Access.

Revision

To follow our recommendation, please upload the revised manuscript files using **the link provided in the decision letter**. Should you need assistance with our manuscript tracking system, please contact Adam Lipkin, our Nature Portfolio Guided OA support specialist, at guidedOA@nature.com.

Revision checklist

- ☐ Cover letter, stating to which journal you are submitting
- ☐ Revised manuscript
- ☐ Point-by-point response to reviews
- ☐ Updated Reporting Summary and Editorial Policy Checklist
- ☐ Supplementary materials (if applicable)

Submission elsewhere

If you choose not to follow our recommendations, you can still take the reviewer reports with you.

Option 1: Transfer to another Nature Portfolio journal
Springer Nature provides authors with the ability to transfer a manuscript within the Nature Portfolio, without the author having to upload the manuscript data again. To use this service, **please follow the transfer link provided in the decision letter.** If no link was provided, please contact guidedOA@nature.com.

> *Note that any decision to opt in to In Review at the original journal is not sent to the receiving journal on transfer. You can opt in to In Review at receiving journals that support this service by choosing to modify your manuscript on transfer.*

Option 2: Portable Peer Review option for submission to a journal outside of Nature Portfolio
If you choose to submit your revised manuscript to a journal at another publisher, we can share the reviews with another journal outside of the Nature Portfolio if requested. You will need to request that the receiving journal office contacts us at guidedOA@nature.com. We have included editorial guidance below in the reviewer reports and open research evaluation to aid in revising the manuscript for publication elsewhere.

Annotated reviewer reports

The editors have included some additional comments on specific points raised by the reviewers below, to clarify requirements for publication in the recommended journal(s). However, please note that all points should be addressed in a revision, even if an editor has not specifically commented on them.

Reviewer #1 information

**Expertise**	This reviewer has expertise in spatial multi-omics.
**Editor's comments**	While we recognize that this reviewer does not fully engage with the text, they pose similar concerns about the technical advance as Reviewers #2-3, which prohibited further consideration from *Nature Methods*.

Reviewer #1 comments

Section	Annotated Reviewer Comments
**Remarks to the Author: Overall significance**	An integrated package to process spatial organization in tissues from multiple spatial omics datasets.
**Remarks to the Author: Impact**	Most methods have previously been highlighted in previous literature. I would suggest *Communications Biology* for this paper.<br>**While we appreciate the reviewer's input, all decisions regarding publication are solely made by editors.**
**Remarks to the Author: Strength of the claims**	Quantitative metrics comparing previous methods seem solid. I suggest this user-friendly comparison to allow other researchers in spatial omics.
**Remarks to the Author: Reproducibility**	The results agree with previously demonstrated spatial organization in previous methods and data analysis techniques. Thus, it sounds reproducible.

Reviewer #2 information	
**Expertise**	This reviewer has expertise in spatial multi-omics.
**Editor's comments**	This reviewer raises several important concerns regarding the readability of the text and requests additional benchmarking to other programs, to further support any technical advance posed by SOTIP. They also note some concerns regarding code availability and deposition.

Reviewer #2 comments	
**Section**	**Annotated Reviewer Comments**
**Remarks to the Author: Overall significance**	Yuan et al. present a new method, SOTIP (Spatial Omics mulTIPle-task analysis), and show its versatility in analyzing a variety of spatial omics datasets including spatially resolved transcriptomics data and proteomics data. In particular, the algorithm performs 3 main analytical functions including spatial heterogeneity quantification (SHN), spatial domain identification (SDM), and differential microenvironment analysis (DME). The authors benchmark against several recently published/pre-printed algorithms (using different algorithms for different datasets and tasks as applicable). The authors also demonstrate some novel biological findings using their algorithm to mine existing data.<br><br>While the method performs well, it is difficult to interpret some of the benchmark studies due to the lack of clarity surrounding the 1) the large number of datasets/modalities/biological systems used in the manuscript and 2) differences between SOTIP and existing algorithms. The manuscript could be more accessible by building into Figure 1 the various spatial omic datasets being used and how they are being used (i.e. for SHN, SDM, or DME). While some of this information is in supplementary tables, it is very difficult to interpret while reading the manuscript. The datasets are often only superficially explained, which also makes it challenging to evaluate the data or how SOTIP performs on different types of data within a modality (for example, single cell spatially resolved transcriptomics vs. Visium). And only some tasks are run on certain datasets while others are not, which again, makes it difficult to interpret the benchmarking and claims of superior performance. Furthermore, SDM is a highly desirable task for spatial omics, but it was difficult to clearly understand the utility of SHN or DME–when one would want to use these tasks and why these tasks might be better employed than other methods–this needs to be better fleshed out.<br>**Concerns about technical advance prohibit further consideration at *Nature Methods*.**

	In general, some novel features of SOTIP that are desirable compared to other algorithms are that it doesn't require pre-determined k for finding spatial domains. It is compatible with proteomics data and 3D data. It also identifies spatial domains more closely resembling ground truth compared to existing algorithms. The tutorials are also extremely useful, although there are some accessibility and clarity issues detailed below.
**Remarks to the Author: Impact**	The development of algorithms for unsupervised analysis of spatial omics data is rapidly growing as more spatial omics data sets come online. There is a need and high interest in improving algorithms for spatial data and SOTIP offers a unique and versatile approach. However, this work may be better suited for a more methods-focused journal. As the manuscript is currently written, it is for a highly specialized spatial biology computational audience (for example, different types of spatial data are not explained well, current algorithms are not described well, and computational terms/metrics are not explained–it is assumed that the reader knows these spatial techniques, algorithms, metrics).<br>**Issues with readability were also noted by Reviewers #3-4.**
**Remarks to the Author: Strength of the claims**	1. As already mentioned, the authors should provide better descriptions of the tasks SOTIP is performing and why and how it compares to other algorithms. For example, there is no explanation of NUCC and IGD, which the authors are benchmarking against. How is SOTIP different from these algorithms?<br>**For the sake of clarity, please ensure that all algorithms are fully explained in the text.**<br><br>2. The authors should include a comparison between squidpy and SOTIP as squidpy uses a similar concept of nodes and distances between nodes to find similarities and dissimilarities.<br>**Please benchmark SOTIP to squidpy or RESEPT (per Point #3, below), for further consideration at *Communications Biology*. Benchmarking to both of these methods would be required for further consideration at *Nature Communications*.**<br><br>3. How does SOTIP compare with RESEPT (https://www.biorxiv.org/content/10.1101/2021.07.08.451210v1) for spatial pattern inference?<br><br>4. A major benefit of BayesSpace is that it can be run across all samples for SDM such that the same clusters are identified across a large sample set. Can SOTIP be implemented across multiple samples or can it only be run tissue section by tissue section?<br>**Please explore the possibility of analysing multiple samples with SOTIP for further consideration at *Nature Communications.***<br>**While *Communications Biology* would encourage you to explore the simulations/scenarios raised in Points #4-5, these could also be mentioned as limitations of SOTIP.**

	5. A major focus in the field has been the integration of single cell and spatial omics data sets. In terms of DME, when relevant single cell data is also available, how does SOTIP outperform algorithms such as Cell2Location (https://www.nature.com/articles/s41587-021-01139-4) that are also able to employ microenvironment analyses using non-negative matrix factorization approaches (see Fig. 4).<br>**Please benchmark SOTIP to Cell2Location for further consideration at *Nature Communications.* This point would be encouraged (but not required) for further consideration at *Communications Biology*.**
**Remarks to the Author: Reproducibility**	**Major comments:**<br>1. As noted, the manuscript lacks clarity and is difficult to read. In general, the authors could improve the clarity of the manuscript in the following ways:<br>a. Shorten the text in all sections and make statements more concise.<br><br>b. Define all acronyms and avoid use of acronyms where possible<br>**We generally recommend that acronyms/abbreviations only be included if they are used >5 times in the main text.**<br><br>c. Remove jargon and better explain in text technical terms that may be unfamiliar to readers such as specific algorithms, spatial-omics data sets, and computational terms.<br><br>d. There are several spelling/grammar issues that should be corrected throughout the manuscript in all sections.<br>**Please carefully proofread the manuscript for clarity. If you would like the assistance of paid editing services to do this, we can recommend our affiliates, Nature Research Editing Service and American Journal Experts. However, please note that use of an editing service is neither a requirement nor a guarantee of publication. Free assistance is also available from our resources page.**<br><br>e.Provide more details in figure legends and explain all arrows.<br><br>f. Parts of results that are better suited for discussion (for example, lines 433-459, 745-773)<br><br>2. There are several issues with code reproducibility:<br>a. Provide code for installation instructions<br><br>b. Unable to render the code on github. For example, these 3 files don't render:<br>• https://github.com/TencentAILabHealthcare/SOTIP/blob/master/SOTIP_analysis/osmFISH_Cortex/SHN_osmFISH_MEwise.ipynb<br>• https://github.com/TencentAILabHealthcare/SOTIP/blob/mast

- er/SOTIP_analysis/MIBI_TNBC/DMA_TNBC.ipynb
- https://github.com/TencentAILabHealthcare/SOTIP/blob/master/SOTIP_analysis/Visium_Zebrafish/SHN_Visium_zebroFISH_A_diff_res.ipynb

c. Visium DLPFC figure 4c does not match the reproducible example on github (https://github.com/TencentAILabHealthcare/SOTIP/blob/master/SOTIP_analysis/Visium_Cortex/SDM_Visium_cortex_best.ipynb)

d. Have the authors provided the exact code to reproduce all the figures in the manuscript?
Please ensure that all code is provided in GitHub, and follow additional guidelines in the Open Research Evaluation at the end of this document to ensure proper transparency and code reporting.

Minor comments:
1.MKT is not defined in the abstract line 46.

2. Provide a clearer and simpler explanation of the term ME (microenvironment).

3. Intro line 68-69 is not clear

4. What do the blue dots along the x-axis in figure 1e represent?

5. Figure 2d legend and figure don't match

6. The result and interpretation of Figure 2f is unclear.

7. Figure 3, subplot ‘L’ missing?

8. The authors should show spatial trajectory graph of sample 151673 in figure 4i

9. Line 492 PAGA has not yet been defined?

10. Figure 5a-c needs more explanation on figure itself and in legend

11. Figure 5d,e which is tumor and which is immune, red or black? Bounded regions are not labels. Arrows are not described.

12. All figures in figure 5 and corresponding supplementary figure should be shown on same axis orientation (e.g. rotate 90 degrees as needed)

13. Line 310- what is meant by clipped?

	14. Figure 6c is not referred to in text<br><br>15. Fig 7, "L" is missing?

Reviewer #3 information

**Expertise**	This reviewer has expertise in single-cell computational biology and biostatistics.
**Editor's comments**	This reviewer finds that SOTIP is well-designed and useful, but raises some concerns regarding the benchmarking and overarching readability (particularly regarding DME analyses).

Reviewer #3 comments

**Section**	**Annotated Reviewer Comments**
**Remarks to the Author: Overall significance**	The manuscript described SOTIP, a suite of software tools for analyzing spatial omics data, where the molecular profiles of individual functional units in the tissue are linked to the spatial location of that unit. For technologies such as Visium and Slide-seq, the functional units are spots or beads used to capture the local RNA molecules, thus the RNAseq data for individual spots or beads are accompanied by their x-y coordinates. There is a strong need for computational tools that can analyze such data. SOTIP is designed to accomplish three main tasks: spatial heterogeneity (SHN) quantification, spatial domain (SDM) identification, and differential microenvironment (DME) analysis. The algorithms were described in Methods; while Results were devoted to reporting performance in various tasks, along with some comparisons with previously published software tools.<br><br>A brief note of the method. For data with N spots and P features, one can use the P-feature vectors to build spot-spot "distance" measures as one of the starting points to explore spatial heterogeneity. In SOTIP, every spot is decomposed as a mixture of t clusters, where the clusters have been predefined, and sometimes referred to as cell types. Thus a second way to build spot-spot distance measures is to use the t-element composition for each spot. SOTIP uses a third measure. For each spot, its neighboring k spots form a "graph" of k edges. The distance between Spot-i and Spot-j is by comparing graph-i and graph-j, where each graph has M edges, from the index spot to its M neighbors, and each of the M+1 nodes is characterized by the K-cluster composition. The rationale is that such a graph represents each spot's microenvironment. This is a good concept and, when used well, can complement each spot's innate properties as measured by the first two options (P features and K-cluster composition). The use of network-based distance measures is part of an original and significant area of research.<br><br>A minor confusion: The graph-to-graph connectivity-based distance is defined

	in line.933. What is confusing is that the same distance seems to also used to calculate edge-distance within each graph (line.954).
**Remarks to the Author: Impact**	The paper has important elements in the development of the algorithms. However, they are not always presented in an easy-to-understand fashion. The exploration of the algorithms may influence the thinking of the field. The claim that the package has better performance is difficult to evaluate, and not likely to have a strong impact.
**Remarks to the Author: Strength of the claims**	1. The descriptions of the algorithms in Methods were not easy to follow, especially with regard to the rationale and the innovation when compared to past efforts. It should be considered to explain the methods first.<br><br>2. Spatial heterogeneity quantification (the first task) is based on the total edge distance among the k neighboring cells. The better performance of the connectivity-based distance is shown in a simulation (Figure 2B). I am not convince because the use of the original P-feature vector would also perform well in the situation in Figure 2B.<br>**Please clarify these tasks (Points #2-5) for further consideration at *Nature Communications* and *Communications Biology*.**<br><br>3. The second task, spatial domain identification, has a major conceptual issue. When each of the N spots has been recoded as a graph with k edges, and each of the k nodes has been recoded as a histogram of t components, the "domain" is found by iteratively clustering the N spots by their connectivity-based distance, resulting in a hierarchical clustering tree. As described in lines.958-985, the spatial relationship of the spots are not used, therefore the "neighboring" spots on the tree may or may not be near each other, to form a domain. It is the reader's guess that the number of clusters will come from a tree-cutting method, say have T clusters by cutting the tree at the level of K branches. But they will not form K domains as the spots within a cluster are likely non-contiguous in the tissue. If spatial proximity is used in the algorithm, it is not described. And if it is indeed used, the relative weight of spatial distance and the connectivity -based distance is the key part of the method. Many existing tools have confronted this issue.<br><br>Besides, clustering of the N spots can be done by using the original P features or the dimension-reduced alternatives, such as the composition of the t clusters. The relative merits of these alternatives need to be better explored, first in simulation, before moving to real data.<br><br>4. The third task, differential microenvironment analysis, requires significant clarification. As described in lines.986-1016, sometimes the comparison seems to be between two different tissue samples, while some other times it seems to be between two different "clusters" within a sample, here the "clusters" seem to be the "domains" found in task-2, not the t spot clusters used in the

	early steps. The analysis is essentially a visualization in EH plot. If there is a test statistics also proposed, it was not easy to find in the text.<br><br>5. The choice of the distance measures discussed in "Connectivity guided minimum graph distance (CGMGD)" is the foundation of SOTIP. The advantages and disadvantages of the distance measures need to be extensively examined.<br><br>6. The Results section focused on examples of application. I did not go through them in detail. There are several difficulties. One is the degree of difficulty of knowing the true signal. Some examples are "easy", such as finding different cortical layers have different spatial properties (Figure 3j-o). What constitutes a relevant improvement of performance is not easy to define.
**Remarks to the Author: Reproducibility**	I did not evaluate the quality of the codes but appreciated that the link to GitHub was provided.<br><br>The simulations will be more helpful if they are described in greater detail: what type of statistical properties was embedded to probe which type of performance.<br>**Please note that Nature Portfolio journals do not have word limits for the Methods section.**<br><br>The terminology can be much improved. "Single cell" was used to refer to spots. The difference between MECN and MEG took repeated reading to understand. Differential analysis of microenvironment can happen on multiple scales. As written it is unclear which scale does the comparison take place.

Reviewer #4 information

**Expertise**	This reviewer has expertise in spatial datasets and microenvironment analyses.
**Editor's comments**	This reviewer also finds that SOTIP is a convincing and useful method, though offers several useful suggestions for simulations to improve the perceived technical advance.

Reviewer #4 comments

**Section**	**Annotated Reviewer Comments**
**Remarks to the Author: Overall significance**	The authors propose a method called SOTIP to perform three main tasks: 1) spatial heterogeneity quantification, 2) spatial domain identification, and 3) differential microenvironment analysis.<br><br>Those tasks are tackled by constructing a MECN graph that uses the gene expression and cell-type information of the neighbourhood. The authors demonstrated their method via both simulation and real data. In general, this is a well-written paper.
**Remarks to the Author: Strength of the claims**	This method is convincing, although I have a few comments.<br><br>**General remarks:**<br>1) In simulation, the tissue structures are fairly simple.<br>Please explain/interpret the SHN values in figure 2b. Are they estimated using the same algorithm? If so, how to interpret the large range of SHN estimated by SOTIP? If not, how to compare across methods?<br><br>Based on simulation 1 (used in Figure 2a), c1 and c2 are indeed two clusters and there does exist spatial heterogeneity, although it is a weak one. NUCC and IGD can clearly identify it. However, the SHN of this boundary estimated from SOTIP is ~200, much smaller than the one for C2 and C3. Is it possible that the statistical power of SOTIP is smaller than another two methods? Similar to Figure 2D, the UMAP of CCNR can confidently split the C1- C5 as five clusters, however, the UMAP of SOTIP cannot, if we don't have prior cluster information. Authors argue this is an advantage that SOTIP preserves the continuous trend in the PC plot, which I'm afraid I have to disagree. In my option, this is a lack of power to cluster confidently.<br><br>For simulation 3 (Figure 2e and 2f), samples 1 and 2 are a clean contrast. C1|C2 in sample 1 is unique compared to sample 2, and C3|C1 does not exist in sample 1. In reality, there could exist C3|C1 in sample 1 as well. For example,

there could be a subtle difference in cell spatial distribution in the different stages of disease progression. But this subtle difference could affect the treatment outcome. In this case, the contrast between samples is not as straightforward as in figure 2e, increasing the noise in identifying spatial heterogeneity. What is the performance of SOTIP in such a case?

Still, for simulation 3, there are equal distances among C1-C3. What is the effect if two clusters are close in PC space (e.g. C1 and C2 Figure 2a), compared to the third cluster (C3 in Figure 2a), but those clusters have different spatial distributions in different samples (eg the distribution of figure 2e).

In all simulations, the number of cells per cluster is almost identical. How the size of a cluster affects the detection of SHN?

In my opinion, comprehensive simulation can provide a good sense on the power and type I error rate of the method.

> **Please clarify these points and include at least one other simulation for further consideration at *Nature Communications* and *Communications Biology*.**

2) Figure 1a, please state what are those red squared boxes standing for? Figure 1e, from left to right, is the degree of spatial heterogeneity increasing or decreasing? In general, please add a more detailed description to the figure captions.

> **This point was raised by other reviewers, and should be addressed for further consideration at *Nature Communications* and *Communications Biology*.**

3) Line 989-990: “but from joining MECNs of two samples”: do you perform PCA, neighbors, umap, Leiden within each sample to get MECN and then combine those two MECN? Or do you combine expression data and spatial data of two samples, then perform PCA, neighbors, umap, Leiden to get a MECN for the combined data? If it is the former one, the same cluster (e.g. Cluster 1) in two samples may stand for different cell types. Do you need to perform cell-type annotation beforehand? If it is the latter, how does the batch effect affect the MEG construction? It was not clear to me how you combined two samples.

4) Figure 6e-i demonstrate the DM in the fibrotic liver, compared to the healthy liver. Is there any DM in the healthy liver? Are they make biological sense?

Minor comments:

5) Too many acronyms and some are quite similar. I suggest having a table/ box listing all the acronyms. This will make the manuscript easier to read.

6) On Lines 145-174 (figure 1a): the MECN is a vector of cell type frequency

	within a cellular neighbourhood. Thus, the definition of the microenvironment is the same as before.<br><br>7) Figure 5: what is the meaning of white errors?<br><br>8) In Figure 7, the choice of interesting EMCN clusters is "bottom-right" of the EH plot. Is there a way to quantify this 'bottom-right'?<br><br>9) Line 352-353: how many cells within a radius of 100 um? Does "the same manner as NUCC" mean that NUCC uses cells within 100um to define a ME, but SOTIP uses 10 nearest neighbouring cells?<br><br>10) Typo on line 426: the mean of SpaGCN is 0.433, instead of 4.33<br><br>11) Supplementary figure 4: a-d, the dots in the scatter plots have different sizes. Please add a legend to explain the meaning of it.<br><br>12) Supplementary figure 9: please explain 'WB'.<br><br>13) Provide a guide for the time, number of CPUs, number of threads etc for each of the analyses. Thus, users can easily set up the required computational resource in their analyses.<br><br>14) Line 712: Keratin+ tumor means keratinocyte and tumour?<br><br>15) Lines 1101, please give sufficient detail about MELD, such that I don't have read the original paper. The same to PAGA.<br><br>16) Lines 1122-1123: I'm afraid I have to disagree that using exactly the same parameter for different models is a fair comparison.<br><br>17) Line 903-910 is not clear to me how to define a MECN. I understand that a MECN is measured per cell, the cell type frequency estimated based on its KNN (10). Or in MEG, each node is a cell, the feature of this node is MECN. The MECN is the cell type frequency estimated based on the cell's KNN.<br><br>18) Table Supp 4, 5 where is a comparison with RESEPT, FICT, ect?
**Remarks to the Author: Reproducibility**	The program on GitHub can be run successfully, and demo data can be downloaded too.

Open research evaluation

Guidelines for Transparency and Openness Promotion (TOP) in Journal Policies and Practices ("TOP Guidelines")

The recommendations and requests in the table below are aimed at bringing your manuscript in line with common community standards as exemplified by the TOP Guidelines. While every publisher and journal will implement these guidelines differently, the recommendations below are all consistent with the policies at Nature Portfolio. In most cases, these will align with TOP Guidelines Level 2.

FAIR Principles

The goal of the recommendations in the table below related to **data or code** availability is to promote the FAIR Guiding Principles for scientific data management and stewardship (*Scientific Data* **3**: 160018, 2016). The FAIR Principles are a set of guidelines for improving 4 important aspects of digital research objects: **F**indability, **A**ccessibility, **I**nteroperability and **R**eusability.

ORCID

ORCID is a non-profit organization that provides researchers with a unique digital identifier. These identifiers can be used by editors, funding agencies, publishers, and institutions to reliably identify individuals in the same way that ISBNs and DOIs identify books and articles. Thus the risk of confusing your identity with another researcher with the same name is eliminated. **The ORCID website** provides researchers with a page where your comprehensive research activity can be stored.

Springer Nature collaborates with the ORCID organization to ensure that your research contributions (as authors and peer reviewers) are correctly attributed to you. Learn more at https://www.springernature.com/gp/researchers/orcid

Data availability

Data Availability Statement

Many journals, including all Nature Portfolio journals, require a Data Availability Statement in the manuscript as a condition of publication. The Data Availability Statement should be as detailed as possible and include accession codes or other unique IDs for deposited data, information about where source data can be found, and specify any restrictions to data access that may apply. At a minimum, the statement should indicate that data are available upon request and explain how data access can be granted. If data access is not possible, the reasons for this must be made clear in the Data Availability Statement.

- More information about the Nature Portfolio data availability policy can be found here.
- Additional information about Data Availability Statements and Springer Nature's data policies are available here.

Mandatory data deposition

Most scientific journals, including all Nature Portfolio journals, require that any newly-generated sequence data must be made publicly available before publication. There are some exceptions allowed for sensitive clinical data, but this should be discussed with the editor. All data must be deposited in a community-approved repository and accession codes/unique IDs must be included within the Data Availability Statement in the manuscript.

Examples of appropriate public repositories are listed below:

- GenBank (all DNA sequence data)
- Sequence Read Archive (high-throughput sequence data)
- Gene Expression Omnibus (Microarray or RNA sequencing data)

More information on mandatory data deposition policies at the Nature Portfolio can be found here. Please also visit this page for a list of approved repositories for various data types.

Other data requests

In line with community standards regarding open research, Springer Nature strongly supports data sharing and believes that all datasets on which the conclusions of the paper rely should be available to readers. We encourage authors to ensure that their datasets are either deposited in publicly available repositories (where available and appropriate) or presented in the main manuscript or additional supporting files whenever possible.

All source data underlying the graphs and charts presented in the main figures must be made available as Supplementary Data (in Excel or text format) or via a generalist repository (eg, Figshare or Dryad). This is mandatory for publication in a Nature Portfolio journal, but is also best practice for publication in any venue.

The following figures require associated source data: Fig 3n-o, 4d, 5f, 5i, 7k-n

Data citation

Please cite (within the main reference list) any datasets stored in external repositories that are mentioned within their manuscript. For previously published datasets, we ask that you cite both the related research article(s) and the datasets themselves. For more information on how to cite datasets in submitted manuscripts, please see our data availability statements and data citations policy.

Citing and referencing data in publications supports reproducible research, by increasing the transparency and provenance tracking of data generated or analysed during research. Citing data formally in reference lists also helps facilitate the tracking of data reuse and may help assign credit for individuals' contributions to research. A number of Springer Nature imprints are signatories of the Joint Declaration on Data Citation Principles, which stress the importance of data resources in scientific communication.

Code availability and citation

Thank you for making your custom code available via Github. Upon publication, Nature Portfolio journals consider it best practice to release custom computer code in a way that allows readers to repeat the published results. **Code should be deposited in a DOI-minting repository such as Zenodo, Gigantum or Code Ocean and cited in the reference list** following the guidelines described in our policy pages (see link below). Authors are encouraged to manage subsequent code versions and to use a license approved by the open source initiative.

- See here for more information about our code availability policies.

Ethics

We believe that research that involves the use of clinical, biomedical or biometric data from human participants must only be carried out with the explicit consent of those whose data are involved. Consent must be obtained without any form of coercion and with participants' explicit understanding of the purpose for which their data will be used.

Because your study includes data from human participants, confirmation that all relevant ethical regulations were followed is needed for publication in any Springer Nature journal, and that informed consent was obtained. This must be stated in the Methods section, including the name of the board and institution that approved the study protocol. Likewise, you should include a statement affirming that studies providing any source data involving live vertebrates complied with all relevant ethical regulations for animal testing and research is necessary. A statement explicitly confirming if the study received ethical approval, including the name of the board and institution that approved the study protocol is also required. The species, strain, sex and age of animals should be included.

Further details about the Nature Portfolio policy can be found here.

Reporting & reproducibility

We believe that research publications should adhere to high standards of transparency and robustness in their methods and results. This, in turn, supports the principle of reproducibility,

which is a foundation of good research, especially in the natural sciences.

The Methods section should contain sufficient detail such that the work could be repeated. It is preferable that all key methods be included in the main manuscript, rather than in the Supplementary Information. Please avoid use of “as described previously” or similar, and instead detail the specific methods used, with appropriate attribution.

Please note that Nature Portfolio journals allow unlimited space for Methods.

Nature Portfolio wishes to improve the reproducibility of the work that we publish. Thus, we ask that you present all key equations in the main manuscript rather than in the supplementary information, and **number these equations sequentially.**.

Statistical reporting

Wherever statistics have been derived (e.g. error bars, box plots, statistical significance) figure legends should provide and define the n number (i.e. the sample size used to derive statistics) as a precise value (not a range), using the wording “n=X biologically independent samples/animals/cells/independent experiments/n= X cells examined over Y independent experiments” etc. as applicable. The figure legends must also indicate the statistical test used. Where appropriate, please indicate in the figure legends whether the statistical tests were one-sided or two-sided and whether adjustments were made for multiple comparisons. For null hypothesis testing, please indicate the test statistic (e.g. F, t, r) with confidence intervals, effect sizes, degrees of freedom and P values noted.

All error bars need to be defined in the figure legends (e.g. SD, SEM) together with a measure of centre (e.g. mean, median). For example, the legends should state something along the lines of “Data are presented as mean values +/- SEM” as appropriate. All box plots need to be defined in the legends in terms of minima, maxima, centre, bounds of box and whiskers and percentile.

For examples of expected description of statistics in figure legends, please see the following:

- https://www.nature.com/articles/s41467-019-11636-5
- https://www.nature.com/articles/s41467-019-11510-4

When describing results as "significant" in the main text, please include details about the statistical test used and provide an exact p-value, rather than a significance threshold.

Please note that statistics such as error bars significance and p values cannot be derived from n<3 and must be removed in all such cases. We also strongly discourage deriving statistics from technical replicates, unless there is a clear scientific justification for why providing this information is important. Conflating technical and biological variability, e.g., by pooling technically replicates samples across independent experiments is strongly discouraged.

To improve reproducibility of your analyses, please provide details regarding your treatment of outliers, as well as detail the methods used for data fitting (along with a rationale for this approach).

Data presentation
Bar graphs should only be used to present counts or proportions. If you are using bar graphs that present means/averages, it is best practice to include individual data points and/or convert the graph to a boxplot or dot-plot. You may wish to refer to this blog post about representing data distribution in plots (particularly for small datasets).
Please ensure that all microscopy images and photographs include a scale bar and this scale bar is defined on the panels or in the figure legends.
Please state in the figure legends how many times each experiment was repeated independently with similar results. This is needed for all experiments, but is particularly important wherever results from representative experiments (such as micrographs) are shown. If space in the legends is limiting, this information can be included in a section titled "Statistics and Reproducibility" in the methods section.
